# Interaction of LARP4 to filamin A mechanosensing domain regulates cell migrations

**DOI:** 10.3389/fcell.2023.1152109

**Published:** 2023-04-24

**Authors:** Zhenfeng Mao, Fumihiko Nakamura

**Affiliations:** School of Pharmaceutical Science and Technology, Tianjin University, Tianjin, China

**Keywords:** mechanotransduction, filamin A, LARP4, FRAP, cell migration

## Abstract

Filamin A (FLNA) is an actin cross-linking protein that mediates mechanotransduction. Force-dependent conformational changes of FLNA molecule expose cryptic binding site of FLNA, allowing interaction with partners such as integrin, smoothelin, and fimbacin. Here, we identified La-related protein 4 (LARP4) as a new FLNA mechanobinding partner. LARP4 specifically interacts with the cleft formed by C and D strands of immunoglobulin-like repeat 21 (R21) which is blocked by A strand of R20 without force. We validated the interaction between LARP4 and FLNA R21 both *in vivo* and *in vitro*. We also determined the critical amino acid that is responsible for the interaction and generated the non-FLNA-binding mutant LARP4 (F277A in human: F273A in mouse Larp4) that disrupts the interaction. Fluorescence recovery after photobleaching (FRAP) of GFP-labeled LARP4 in living cells demonstrated that mutant LARP4 diffuses faster than WT LARP4. Proximity ligation assay (PLA) also confirmed their interaction and disruption of actin polymerization diminishes the interaction. Data mining of RNAseq analysis of LARP4 knockdown (KD) HEK293T cells suggested that LARP4 is involved in morphogenesis and cell motility. Consistent with this prediction, we found that KD of LARP4 increases cell migration speed and expression of the F277A mutant LARP4 in LARP4-KD cells also leads to a higher cell migration speed compared to WT LARP4. These results demonstrated that the LARP4 interaction with FLNA regulates cell migration.

## Introduction

Mechanotransduction is a cellular process that cells convert mechanical stimuli from their extracellular environment or intracellular myosin-generated forces into biochemical signals to induce cellular responses and gene expression (Broders-Bondon et al., 2018; Chighizola et al., 2019; Stewart et al., 2020). A growing body of evidence has demonstrated that mechanotransduction plays a critical role in cell proliferation, division, adhesion and differentiation (Calvo et al., 2013; Gudipaty et al., 2017; Petridou et al., 2017; Pinheiro and Bellaϊche, 2018; Yamashiro et al., 2020), and defects in cellular mechanotransduction pathways are implicated in many diseases (Wozniak and Chen, 2009; Chang et al., 2019; Panciera et al., 2020; Tsata and Beis, 2020). Previous studies found that filamin A (FLNA) is a mechanosensor and mechanotransducer that senses and converts mechanical forces into biochemical signals and thus mediates the mechnotransduction (Ehrlicher et al., 2011; Razinia et al., 2012; Nakamura et al., 2014). FLNA is a F-actin cross-linking protein and acts as a scaffold for a wide range of molecules, including intracellular signaling molecules, adhesion molecules, ion channels and transcription factors (Stossel et al., 2001; Popowicz et al., 2006; Zhou et al., 2007; Nakamura et al., 2011). FLNA mutations related to various human diseases including peri-ventricular nodular heterotopia, familial cardiac valvular dystrophy, skeletal dysplasias and thrombocytopenia (Nurden et al., 2011; Clapham et al., 2012; van der Werf et al., 2013; Fernández et al., 2018; Azakli et al., 2019; Toomer et al., 2019).

FLNA is a homodimer protein with two 280 kDa subunits, each of which consists of an N-terminal actin-binding domain followed by 24 immunoglobulin-like (Ig) repeats (R). Two flexible hinges separate the subunit into two rods. Repeat 1–15 is referred to as rod 1 and 16–23 as rod 2. The repeat 24 is responsible for the dimerization. FLNA’s rod 2, where interactions of FLNA with its over 150 binding partners take place, has a unique geometry and a compact structure that responds to mechanical force through conformational changes to regulate its partner interactions (Nakamura et al., 2011; Rognoni et al., 2012; Ruskamo et al., 2012; Chen et al., 2013). To identify a binding partner that specifically interacts with the mechanosensitive binding site, we recently established a method by means of stable isotope labeling by amino acids in cell culturing (SILAC)-based proteomics using R21-23 as affinity ligand, and identified several potential new mechanobinding proteins such as smoothelin and fimbacin (Wang J. and Nakamura F. 2019; Wang L. and Nakamura F. 2019).

Here, we identified LARP4, La-related protein 4, as a new mechanobinding partner of FLNA and characterized the interaction. LARP4 is an RNA binding protein that binds to the poly-A tract of mRNA molecules and mainly localized in the cytosol while partially localized in stress granules in response to arsenite treatment (Yang et al., 2011). Previous studies showed that overexpression of LARP4 resulted in increased mRNA stability whereas knockdown (KD) of LARP4 caused a reduction in translation, indicating that LARP4 promotes mRNA stability (Yang et al., 2011). Furthermore, recent studies reveal that LARP4 regulates migration and invasion of cancer cells. KD of LARP4 induces elongated phenotype in the prostate cancer cells and increased cell migration, while overexpression of LARP4 decreased the elongation (Bai et al., 2011; Seetharaman et al., 2016). Moreover, more than 224 mutations in LARP4 have been identified in various cancer types according to the catalogue of somatic mutations in cancer (COSMIC) reports (https://cancer.sanger.ac.uk/cosmic/gene/analysis?ln=LARP4). However, their genotype-phenotype relationships are not known.

In the present study, we validated the interaction between LARP4 (Human LARP4 and mouse Larp4) and FLNA R21 both *in vivo* and *in vitro*. Immunofluorescence microscopy demonstrated co-localization of FLNA and LARP4 in HEK293A and human skeletal muscle (hsSKM) cells. Using the *in vitro* site-directed mutagenesis system, we determined the critical amino acid that is responsible for the interaction and generated the non-FLNA-binding mutant LARP4 (F277A). FRAP analysis of GFP-LARP4 expressed in living cells demonstrated that mutant LARP4 diffuses faster than WT LARP4. PLA also detected the interaction between FLNA and LARP4 in tissue culture cells and demonstrated that disruption of actin polymerization diminishes the interaction. Consistent with RNAseq data of LARP4-KD HEK293T cells, KD of LARP4 significantly increases cell migration speed and add-back of the mutant LARP4 into LARP4-KD cells also led to a higher cell migration speed compared to WT LARP4. Collectively, these results demonstrated that interaction of LARP4 to FLNA mechanosensing domain regulates cell migrations.

## Materials and methods

### Antibodies and reagents

Rabbit polyclonal anti-LARP4 antibody (16529-1-AP) was obtained from Proteintech (China). Mouse monoclonal anti-GFP antibody (MA5-15256) was purchased from Thermo Fisher. Rabbit anti-GFP antibody (AE011) was acquired from Abclonal (Wuhan, China). Goat anti-mouse IgG (H + L)-horseradish peroxidase (HRP) (172-1011) and Goat anti-rabbit IgG (H + L)-HRP (172-1019) were from BioRad. Mouse monoclonal anti-FLNA (sc-71118) was from Santa Cruz. Mouse monoclonal anti-GAPDH antibody (MA1-16757) and mouse monoclonal anti-6X His antibody (MA1-21315) were purchased from Thermo Fisher. Mouse monoclonal anti-*β*-Actin antibody (A00702-100) was purchased from GenScript. Alexa Fluor Plus 488 and 594, and Hoechst 33,342 were purchased from Thermo Fisher Scientific. Glutathione-Sepharose (L00206) was purchased from GE Healthcare. Ni-NTA resin (L00250-25) was obtained from Genscript. Streptavidin (Z02043-5, Genscript) was immobilized on NHS-activated Sepharose 4 Fast Flow beads (GE Healthcare) at 2 mg streptavidin/1 mL beads according to the manufactures’ protocol.

### Plasmid construction

Human and mouse LARP4 cDNA (UniProt Accession ID Q71RC2 and Q8BWW4, respectively) were amplified by PCR using 5′ primer CCC​AAG​CTT​CCA​TGT​TGC​TTT​TCG​TGG​AGC​AGG​TAG​C, 3′ primer CGC​GGA​TCC​TTA​CTT​TGG​TGA​TCT​GGG​TGG​CAC​ATA​TTG, and 5′ primer TAC​GTC​GAC​TCA​TGT​TGC​TCT​TCG​TTG​AGG​TGA​C, 3′ primer CGA​GCG​GCC​GCT​TAC​TTT​GGT​GAT​CTG​GGT​GGC, HEK293A and MEF cDNA libraries as templates and ligated into pAcGFP-C1 (Clontech) vectors at *Hind* III/*BamH* I and *Sal* I/*Not* I sites, respectively. Site-directed mutagenesis was performed using the Q5^®^ site-directed mutagenesis kit (NEB). For bacterial expression, fragments of Larp4 were amplified by PCR and ligated into pGEX4T-1 (GE Healthcare) vectors. pSBP (streptavidin-binding protein, GHVVEGLAGELEQLRARLEHHPQGQ)-C1 was constructed by ligating annealed double strand DNA of *Age* I-SBP (CCG​GTA​TGG​GCC​ACG​TGG​TGG​AGG​GCC​TGG​CCG​GCG​AGC​TGG​AGC​AGC​TGA​GAG​CCA​GAC​TGG​AGC​ACC​ACC​CCC​AGG​GCC​AGA​GAG​AGG) and SBP-*BamH* I (GAT​CCC​TCT​CTC​TGG​CCC​TGG​GGG​TGG​TGC​TCC​AGT​CTG​GCT​CTC​AGC​TGC​TCC​AGC​TCG​CCG​GCC​AGG​CCC​TCC​ACC​ACG​TGG​CCC​ATA) into pFLAG-C1 (Clontech) vector digested with *Age* Ⅰ/*BamH* Ⅰ. pSBP-FLNA (WT and Del41) vector was constructed by two steps. First, 5’ *BamH* I/*Sal* I fragment digested from PCR product amplified from actin-binding domain of FLNA was ligated into pSBP-C1 digested with *BamH* I/*Sal* I. Second, *Sal* I/*Not* I fragment digested from pFLAG-FLNA (WT and Del41) was ligated into the pSBP-FLNA N-terminal fragment. pET23-HTb-eGFP-R21-22 and R1-2 were constructed using pET23-HTb-EGFP bacteria expression vector by PCR.

### Protein expression and purification

Bacterial expression was performed with BL21(DE3) Star or C41 (NEB) grown in LB medium in accordance with the manufacturer’s protocol. Briefly, protein expression was induced for 2 h at 37°C in the presence of 0.8 mM isopropyl β-D-thiogalactopyranoside (IPTG). The bacterial cells were harvested by centrifugation (4,000×g, 10 min, 4°C), washed with PBS. The cells were disrupted by sonication (30s on/30s off for 5 min at 25% amplitude by Ultrasonic Processor, Cole-Parmer) in 20 mM sodium phosphate, pH 8.0, 5 mM EGTA, 0.1% Tween20 for GST-tagged protein (20 mM sodium phosphate, pH 8.0, 300 mM NaCl, 20 mM imidazole, pH 8.0 for His-tagged protein) and soluble proteins were then separated by centrifugation (15,000×g for 20 min at 4°C). GST- and His-tag fusion proteins were purified using glutathione-Sepharose and Ni-NTA beads, respectively, in accordance with manufacturer’s protocol (GE Healthcare).

### Coomassie brilliant blue staining

Purified GST-tagged and His-tagged proteins were subjected to 9% or 15% SDS-PAGE gels followed by Coomassie brilliant blue R-250 (CBB) staining. The gels were stained by CBB solution (0.25% (w/v) CBB, 30% (v/v) methanol, 10% (v/v) acetic acid) at room temperature for 30 min using a shaker (speed: one turn/1–2 s). Redundant dye was removed by immersing the gels in Destaining solution (30% (v/v) methanol, 10% (v/v) acetic acid) at room temperature for 2 h or overnight.

### Affinity ligand

Using pFASTBAC-FLNA vector (Nakamura et al., 2002) as the template, FLNA repeats 21-22 (test) and 1-2 (negative control) were cloned into pGEX4T-HT vector by PCR. The vectors were transformed into *E. coli* C41 (NEB) and protein expression was induced by 0.4 mM IPTG for 2 h. The proteins were affinity purified using glutathione beads and the GST-His tag was cleaved off by tobacco etch virus (TEV) protease. Purified FLNA repeats 21-22 and 1-2 were covalently coupled to NHS-activated Sepharose 4 Fast Flow beads (GE Healthcare) at 10 mg per 1 mL of the beads in PBS for 2 h at room temperature. The non-reacted groups of the beads were blocked with 0.1 M Tris-HCl pH 8.0 for 2 h at room temperature, equilibrated with TTBS (50 mM Tris-HCl, pH 7.4, 150 mM NaCl, 1% TritonX-100, 1 mM EGTA, 1 mM β-mercaptoethanol), and stored at 4°C.

### Cell culture, transfection, and SILAC labeling

Hela and human skeletal muscle (hsSKM) cells were purchased from ATCC. HEK293A cells were purchased from Thermo Fisher. These cells were grown in DMEM (Biological industries, Israel) supplemented with 10% FBS (Biological industries, Israel) and 1% penicillin-streptomycin. Cells were maintained at 37°C and 5% CO_2_. Cells were transfected with polyethylenimine (PEI) or LipoGene 2000 Star Transfection Reagent (US Everbright). Mouse embryonic fibroblast (MEF, ATCC) cells were grown for at least six generations in Dulbecco’s modified Eagle’s medium (DMEM) for SILAC (Thermo Fisher) supplemented with L-lysine and L-arginine (light) or L-lysine-13C6 and L-arginine-13C6, 15N4 (heavy) (Thermo Fisher) as previously described (Wang J. and Nakamura F. 2019). 50 mg of each amino acids was added into every 500 mL DMEM for SILAC.

### Affinity purification for mass spectrometry

Labeled MEF cells were grown on 100 mm tissue culture dishes at about 90% confluency and lysed in 1.5 mL of ice-cold TTBS solution supplemented with complete EDTA-free protease inhibitor cocktail (P8340, Biological Industries, Israel), and 2 μM latrunculin B (428,020, Thermo Fisher). Debris was pelleted at 16,000 × g at 4°C for 20 min, and the supernatant was incubated with 20 μL of the affinity beads for 2 h at 4°C. The beads were washed three times with 800 μL of TTBS solution, bound protein was eluted with LDS sample buffer containing 50 mM dithiothreitol (DTT), and eluates from both samples (heavy and light) were pooled. The samples were resolved on precast PAGE gels (Novex 4%–20% Tris-Gly gel; Thermo Fisher), stained with colloidal Coomassie (SimplyBlue SafeStain; Thermo Fisher), and analyzed by liquid chromatography–MS/MS-based quantification. For that, the lane was cut into 7 slices, all of which were subsequently subjected to in-gel tryptic digestion.

### Mass spectrometry and data analysis

Mass spectrometry, data analysis, and database searches were performed as previously described (Cox et al., 2009). Briefly, digested peptides were analyzed by LC-MS/MS on LTQ-OrbitrapXL hybrid mass spectrometer (Thermo Scientific, San Jose, CA). Protein identification and relative quantification was performed using Andromeda and MaxQuant (version 1.3.0.5) (Cox et al., 2011). The subsequent bioinformatics and statistical analyses were performed with Perseus 1.4.1.3 (http://www.maxquant.org).

### *In vitro* binding assay

GST-Larp4 protein were immobilized on glutathione beads (30 μL) in TBS-0.1% Tx and incubated with purified His-eGFP-FLNA R21-22 for 1 h at room temperature. The beads were sedimented and washed with TBS-Tx buffer three times. Bound proteins were solubilized in SDS sample buffer and separated by a 9% Tris-glycine gel. Immunoblotting was performed using an anti-GFP antibody. To identify Larp4 binding site on FLNA, purified His-tag FLNA fragments were incubated with GST-Larp4 immobilized on glutathione beads. Bound FLNA fragments were detected by western blotting using anti-His antibody.

### Pulldown assay using GST-FLNA fragments

HEK293A cells transfected with pAcGFP-Larp4 were solubilized in TTBS supplemented with complete EDTA-free protease inhibitor cocktail on ice. The lysate was incubated with 30 μL of glutathione beads coated with GST-His-FLNA fragments (R1-2 and R21-22) and incubated for 2 h at 4°C. The beads were sedimented and washed with ice-cold TBS-0.1% Tx (50 mM Tris-HCl, pH 7.4, 150 mM NaCl containing 0.1% Triton X-100) three times. Bound proteins were solubilized in SDS sample buffer and separated by a 9% Tris-glycine gel. Immunoblotting was performed using anti-GFP antibody.

### Immunoprecipitation of endogenous LARP4 and SBP-FLNA

HEK293A cells transfected with SBP-FLNA (WT or Del41) were solubilized in TTBS supplemented with complete EDTA-free protease inhibitor cocktail on ice. The lysate was incubated with 30 μL of streptavidin coated beads and incubated for 2 h at 4°C. The beads were incubated with the hsSKM and MEF cell lysate respectively for 1 h at 4°C and finally the beads were sedimented and washed with ice-cold TBS-0.1% Tx (50 mM Tris-HCl, pH 7.4, 150 mM NaCl containing 0.1% Triton X-100) three times. Bound proteins were solubilized in SDS sample buffer and separated by a 9% Tris-glycine gel. Immunoblotting was performed using anti-LARP4 antibody, and streptavidin-HRP (ab7402, Abcam).

### Western blotting

Cell lysates in SDS sample buffer were loaded onto SDS-PAGE gel. Separated proteins were transferred to nitrocellulose membrane and blocked with blocking buffer (5% non-fat milk powder in TBST (20 mM Tris-HCl, pH 7.4, 150 mM NaCl, 0.05% Tween 20). Primary antibodies were prepared in this blocking solution and membranes were incubated overnight at 4°C. The membrane was washed in blocking buffer and incubated with HRP-conjugated secondary antibodies in TBST for 1 h at room temperature. The membrane was washed and developed with the HRP substrate (WesternBright ECL, Advansta).

### Immunofluorescence microscopy

Cells were plated on a poly-lysine- or fibronectin-coated cover glass, transfected with a plasmid, fixed with 4% formaldehyde in PBS for 20 min, rinse in PBS, permeabilized with 0.5% Triton X-100 in TBS for 5 min, rinse in TBS-0.1% Tx, blocked in 2% BSA in TBS-0.1% Tx, and incubated with primary antibodies for 2 h. After several washes with TBS-0.1% Tx, the cells were incubated with secondary antibodies (Thermo Fisher), washed with TBS-0.1% Tx, and mounted with mounting media (Spring Bioscience). Cells were imaged on Leica SP8 X confocal microscope. Images were processed using ImageJ software (NIH). Colocalization was analyzed using Colocalization Finder plug-in.

### Knock-out of LARP4 in tissue culture cells

LARP4 KO HEK293A cells were generated by delivery of Cas9 and target-specific guide RNAs (gRNAs). Oligos encoding the gRNAs for LARP4 were designed using CRISPick (https://portals.broadinstitute.org/gpp/public/analysis-tools/sgrna-design) and the selected LARP4-specific gRNAs sequence, 5′-TAG​ACC​GAG​TAC​TGT​TGG​TG-3′ and 5′-TTG​CGG​CGG​CGG​GAA​CGA​TT-3′, were cloned into *BbsI* digested pX330-U6-Chimeric_BB-CBh-hSpCas9 (Addgene plasmid ID: 42,230). Px330-LARP4 plasmids were transfected into HEK293A cells using LipoGene2000 Star transfection reagent (US Everbright) according to manufactures’ protocol. Briefly, HEK293A cells were seeded into a 24-well plate. After 24 h that the cells reached 60%–70% confluent, 1 μg px330-LARP4 plasmid was added to the well in the presence of LipoGene2000 Star transfection reagent. 72 h post-transfection, cells were then separated as single cells into a 96-well plate by serial dilution for another 7 days. Individual clones were expanded, and LARP4 protein expression was examined by immunoblotting.

### Cell migration assay

For random cell migration, HEK293A cells and LARP4-KD cells were seeded at 10^4^ cells per well on 6-well plates. 24 h later, pAc-GFP-LARP4 WT and F277A plasmids were transfected into LARP4-KD cells using LipoGene2000 Star transfection reagent (US Everbright) according to manufactures’ protocol. After a further 24 h, images were acquired for 16 h at 1 frame/10 min at 37C using a ×10 Plan FL objective on an EVOS^®^ FL Auto time lapse microscope with a monochrome and color camera. Cells were tracked using ImageJ (Plugin: Manual tracking) to obtain migration speed (μm/min). Cells that died, divided, or moved out of the frame were excluded from the analysis and tracking. The path of each cell was obtained as a track using ImageJ (Plugin: Chemotaxis tool).

### Fluorescence recovery after photobleaching (FRAP) assay

HEK293A cells expressing WT or mutant GFP-LARP4 were captured onto glass bottom dishes coated with poly-L-lysine. Cells were imaged in growth medium without phenol red, 50 mM HEPES, and 1.5% FBS using a Leica SP8 X confocal microscope. FRAP was performed as described previously (Wang L. and Nakamura F. 2019). Briefly, the regions of interest (3.00 μm × 3.00 μm) were photobleached for ∼9 s at maximum 514-nm laser power. Subsequently, time lapse images were collected at 2% laser power until the bleached signal reached a stable level. FRAP curves from four independent trials with five cells per trial were derived by fitting the normalized fluorescence at each time point versus time into a one-phase association model plugged into the Prism software. F_max_, which represents the mobile fraction of the molecule in the bleached region, and τ_½_, which is the time to recover half of the maximum fluorescence and is inversely correlated to the diffusion coefficient, were derived from this curve.

### FLNA domain specific antibodies

DNA encoding human FLNA R1, R22 and R23 were cloned into pET23-HTa plasmid and expressed in *E. coli* BL21 cells, respectively. The cell pellets were lysed by sonication in 20 mM sodium phosphate, pH 8.0, 300 mM NaCl, 20 mM imidazole, pH 8.0, and centrifuged at 150,00×g for 20 min at 4°C to separate the soluble and insoluble fractions of the cells. The fusion protein was purified by high affinity Ni-NTA (Genscript) with 300 mM imidazole. After cleavage of the His tag with TEV protease, the protein was purified from the cleaved tag, the His-tagged TEV, and any undigested product by gel filtration using Enrich SEC650 10 × 300 column (BioRad) in 20 mM sodium phosphate (pH 8.0). Then 2 mg of the purified protein was used to immunize rabbits to gain antiserum (ABclonal). The serum was purified by FLNA R1, R22 and R23 protein coated NHS-activated Sephrose (GE Healthcare), respectively and the antibody was eluted by 10 mM acetate buffer (pH 2.5). Eluted antibody was concentrated with a 50 K MWCO centrifugal filter (Amicon). The specificity and potency of polyclonal antibody were evaluated by Western blot.

### Proximity ligation assay

#### Preparation of proximity probes

Polyclonal antibodies (FLNA R1, R22, R23 and LARP4) were covalently coupled to oligonucleotides (Supplementary Table S1). For each conjugation, 50 μg of antibodies in 55 mM phosphate buffer, 150 mM NaCl and 20 mM EDTA (pH 7.2) was activated by addition of a 10-fold molar excess of dibenzyl cyclooctyne NHS ester (DBCO-NHS ester; Jena Bioscience), freshly dissolved in DMSO (Solarbio) and incubated at room temperature for 30 min. Thereafter, the activated antibodies were purified from DBCO-NHS using the 50 K MWCO centrifugal filter (Amicon) that had been equilibrated with 55 mM phosphate buffer, 150 mM NaCl and 20 mM EDTA (pH 7.2). The activated antibodies were then mixed with a 2.5-fold molar excess of the respective azide-modified oligonucleotides, and incubated overnight at 4°C. Excess oligonucleotides were removed from the reactions using the 50 K MWCO Amicon Ultra-4 centrifuge tubes that had been equilibrated with 5 mM EDTA in PBS at 4,000 g for 5 min for 5 times. The average number of conjugated DBCO molecules per antibody ( $n^{D-IgG}$ ) was measured and quantified by absorption spectroscopy, using the following equation: $n^{D-IgG}=$ $\frac{c^{D}}{c^{IgG}}$ ( $c^{D}$ , where the DBCO ( $c^{D}$ ) and antibody ( $c^{IgG}$ ) concentration were obtained by $c^{D}=$ $\frac{A_{309}}{\varepsilon_{309}^{D}}$ and $c^{IgG}=$ $\frac{A_{280}^{c}}{\varepsilon_{280}^{IgG}}$ , respectively. The molar extinction coefficients of the DBCO and IgG antibody are at 309 nm (12,000 M^−1^ cm^−1^) and 280 nm (204,000 M^−1^ cm^−1^), respectively. $A_{309}$ is the absorption value of the sample at 309 nm and $A_{280}^{c}$ is the absorption value of the sample corrected by the absorption contribution of DBCO at 280 nm. $A_{280}^{c}$ is calculated by $A_{280}^{c}=A_{280}-\left(A_{309}∙f\right)$ where the $A_{280}$ is the absorption value of the sample at 280 nm and *f* the correction factor of DBCO at 280 nm (*f* = 1.1).

### Application of probes *in situ*

HEK293A cells were seeded into a 96-well plate at a density of approximately 20,000 cells/cm^2^ in DMEM with 10% FBS supplemented with penicillin and streptomycin and grown for 1–2 days until 70%–80% confluent. Cells were fixed with 3.7% formaldehyde for 20 min at room temperature and permeabilized with 0.5% Triton X-100 in PBS for 5 min at room temperature and washed twice with 0.05% Tween-20 in TBS. The cells were blocked by treatment with 250 ng/mL BSA (Genview), 50 ng/mL RNase A (TakaRa), 5 mM EDTA, 11 ng/mL poly(A) (Sigma) and 0.05% Tween-20 in TBS for 2 h at 37°C before overnight incubation at 4°C with 7.5 ng/mL proximity probes, 7.5 ng/mL poly(A), 2.5 mM cysteine (Solarbio), 250 ng/mL BSA and 0.05% Tween-20 in TBS with 5 mM EDTA. Two connector oligonucleotide probes at 125 nM in 10 mM Tris-acetate (pH 7.5), 10 mM magnesium acetate, 50 mM potassium acetate, 0.05 U/mL T4 DNA ligase (NEB), 250 mM NaCl, 250 ng/mL BSA and 0.05% Tween-20 in H_2_O were applied to the cells and ligated the probes to form circles using as templates the two oligonucleotides attached to the antibodies. Ligations were performed at 37°C for 1.5 h. The ligated circles were amplified with 0.125 U/mL phi29 DNA polymerase (NEB) in 50 mM Tris-HCl (pH 7.5), 10 mM MgCl_2_, 10 mM (NH_4_)_2_SO_4_, 250 mM dNTPs, 250 ng/mL BSA and 0.05% Tween-20 at 37°C for 1.5 h. The single-stranded RCA products were detected by hybridization with 10 nM fluorescence-labeled probe in 2X SSC, 7.5 ng/mL poly(A), 250 ng/mL BSA and 0.05% Tween-20 for 1 h at 37°C. The nucleus was stained with Hoechst 33,342.

PLA spots were identified and cells were manually counted based on positive Hoechst staining and quantified using the software based on red signal intensity and spot size. PLA interactions per cell were calculated by dividing the PLA spots by the total number of cells in each region of interest. PLA interactions per cell were plotted, and a *t*-test was performed using GraphPad Prism software version 9 (GraphPad Software Inc., La Jolla, CA).

### RNA-seq data acquisition and analysis

The RNA-seq data of LARP4 knockdown (KD) HEK293T cells are publicly available on https://www.ncbi.nlm.nih.gov/bioproject/PRJNA580238 (and https://www.ncbi.nlm.nih.gov/geo/query/acc.cgi?acc=GSE139548).

SRR data were downloaded from https://www.ncbi.nlm.nih.gov/Traces/study/?acc=PRJNA580238&o=acc_s%3Aa. The downloaded fastq data were processed via Trim Galore 0.6.6. Quantification and differential expression analysis were performed using the pseudo-alignment program Salmon (Patro et al., 2017) and iDEP (integrated Differential Expression and Pathway analysis) online tools (http://bioinformatics.sdstate.edu/idep/).

**Statistics.** Data are mean ± S.E.M. All experiments were performed at least three times independently. All image analysis was performed by operators who were blinded to the treatments administered. For significant results, ∗∗∗∗ denotes *p* < 0.0001, ∗∗∗*p* < 0.001, ∗∗*p* < 0.01, ∗*p* < 0.05, and ns *p* > 0.05.

## Results

### SILAC-based proteomics identified LARP4 as a new filamin A binding partner

**FIGURE 1:**
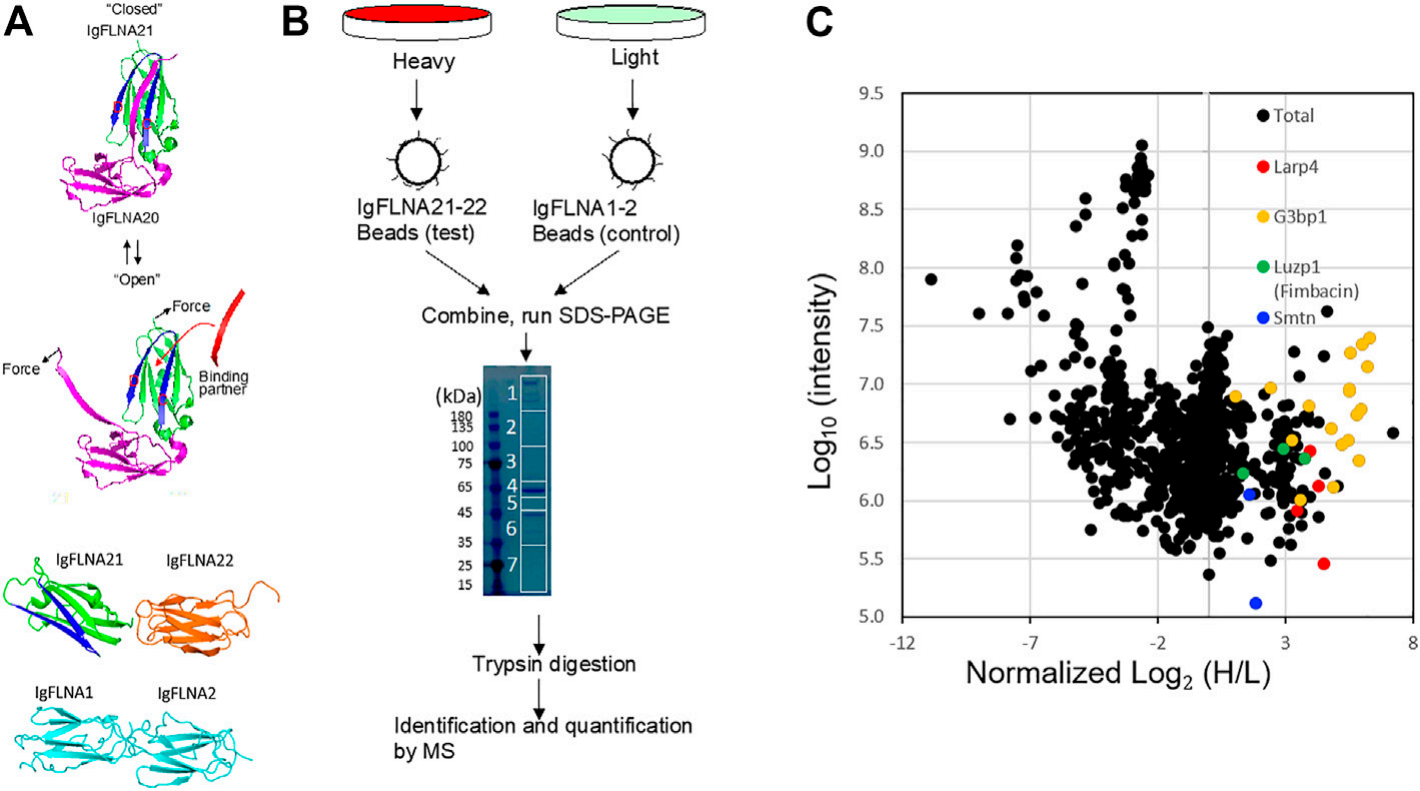
SILAC-based proteomics of FLNA-binding partner. **(A)** The CD cleft (blue) opens for partner interaction in force-dependent manner. FLNA domains used for affinity purification of mechanically-regulated FLNA-binding partner. IgFLNA1-2 was used as a negative control. **(B)** Schematic representation of the SILAC-based mass spectrometry experiments. **(C)** Standard scatterplots with normalized Log_2_ (H/L) ratios/Log_10_ Intensities (control versus test) highlighting the distribution of quantified proteins in each MS screening.

Several studies have provided evidence that the interaction partner of FLNA forms an additional *β*-strand next to the strand C of the FLNA domain and simultaneously interacts with the hydrophobic groove between the strands C and D, called the CD face (Kiema et al., 2006; Nakamura et al., 2006; Lad et al., 2008; Ithychanda et al., 2009). However, the CD faces of R21 are covered by the strand A of R20 (Lad et al., 2007) (Figure 1A). Removal of R20 by mechanical forces exposes the CD face of R21 (Ehrlicher et al., 2011). To identify a binding partner that specifically interacts with the mechanosensitive binding site, we previously performed the SILAC-based quantitative proteomics followed by mass spectrometry (MS) analysis using FLNA R21-22 as affinity ligand and cell lysate of mouse embryonic fibroblast (MEF) (Wang J. and Nakamura F. 2019). FLNA R1-2 was used as a negative control because the structurally known binding partners that bind to R21 do not interact with R1-2 (Figure 1B). Of note that R22 was attached to R21 to make the ligand spatially more accessible for a binding protein on affinity beads. These proteins were expressed as GST-His fusion proteins in *E. coli*, purified by affinity chromatography, cleaved from GST-His tag, and covalently attached to NHS-Sepharose beads. Using these affinity beads, we pulled several proteins from the MEF cell lysate that specifically bind to R21-22 (Figure 1B).

The MS data revealed over 120 potential binding partners that interact with FLNA R21-22 (Feng et al., 2023). Among these proteins are known FLNA binding partners such as smoothelin and fimbacin (Figure 1C). Since we detected multiple peptides of Larp4 with over 2-fold H/L ratio (Figure 1C), we further investigated if Larp4/LARP4 is a new mechano-binding partner of FLNA.

### LARP4 specifically binds to FLNA R21 mechanosensing site

**FIGURE 2:**
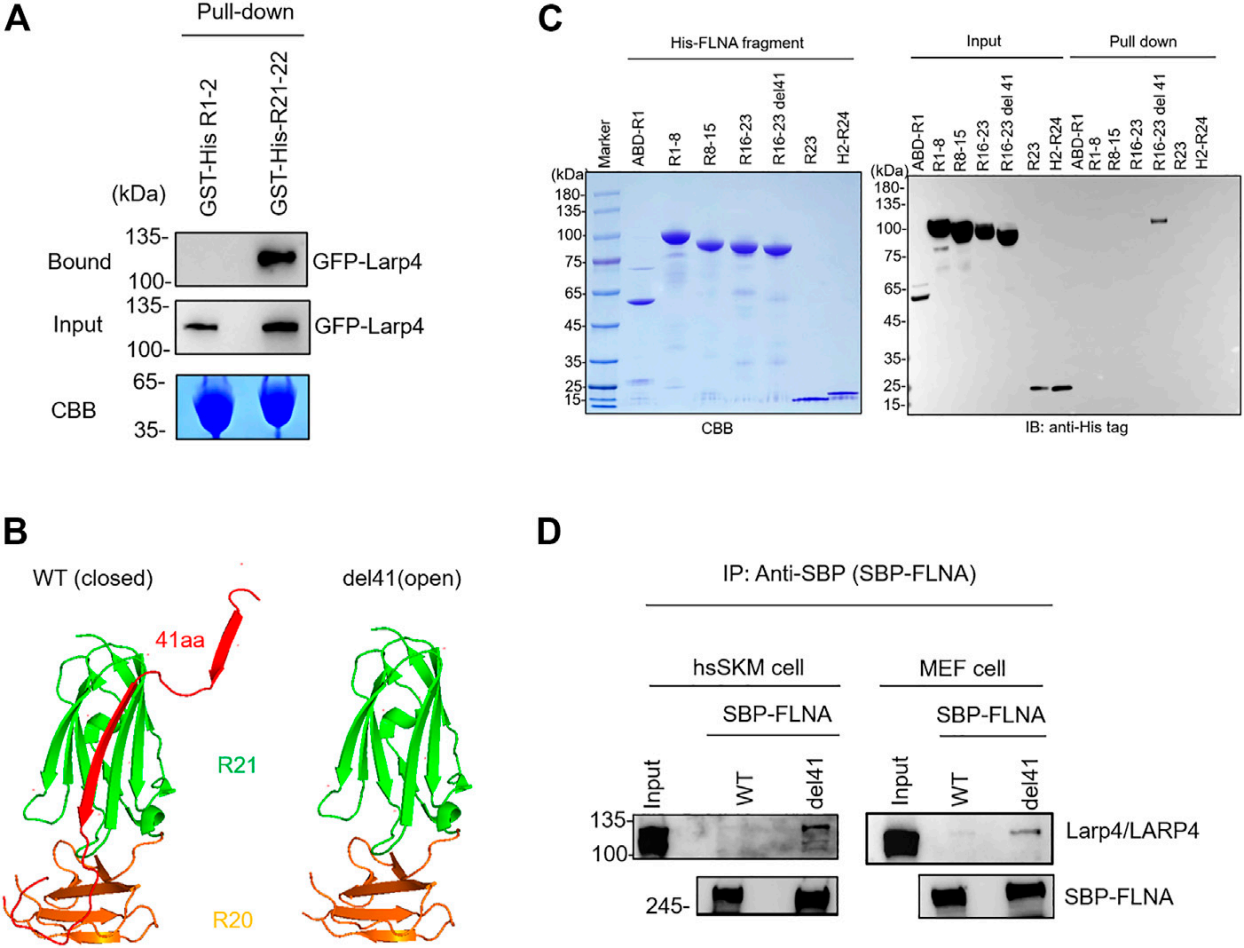
Selective interaction of LARP4 with open FLNA. **(A)** GFP-Larp4 was expressed in HEK 293A cells and the expressed protein was pulled down with purified GST-His-FLNA fragments. CBB staining showed 5 μg of the purified GST fusion protein loaded to the 9% SDS-PAGE gel. Bound GFP-Larp4 was detected by western blotting using rabbit anti-GFP antibodies. **(B)** The CD face of R21 is blocked with strand A of R20 in WT FLNA (left). Deletion of 41 amino acid residues (del41) constitutively exposes the cryptic binding site (right). **(C)** Mapping of Larp4-binding site on FLNA. Purified His-tagged FLNA fragments were pulled down with GST-Larp4 immobilized on glutathione beads. CBB staining showed 2 μg of the purified His-tagged FLNA fragments protein loaded to the 4%–12% SDS-PAGE gel. The lower bands of the input proteins are indicative of degradation during expression in bacteria. Black arrow indicates the GST-Larp4. Bound His-tag FLNA fragments were detected by western blotting using anti-His-tag antibody. **(D)** SBP-FLNA (WT and del41) was expressed in HEK293A cells and immobilized on streptavidin-beads. The beads were incubated with lysates of hsSKM (left) or MEF (right) cell and bound Larp4/LARP4 was detected by western blotting using anti-LARP4 antibodies. CBB: Coomassie Brilliant Blue. IB: Immunoblot. IP: Immunoprecipitation.

To confirm direct interaction of mouse Larp4 to FLNA, GST-His-FLNA R21-22 immobilized on glutathione beads were incubated with lysate of cells expressing green fluorescent protein (GFP)-tagged Larp4. After washing unbound proteins, bound protein was detected by western blotting against GFP. As expected, GFP-Larp4 was co-precipitated with FLNA R21-22 but not with R1-2 (Figure 2A). Furthermore, purified His-FLNA R21-22 were pulled down with GST-Larp4 immobilized on glutathione beads (Supplementary Figure S1). These results clearly demonstrated that mouse Larp4 protein directly interacts with FLNA R21-22. To map the interaction domain of Larp4 on FLNA, His-tagged FLNA fragments (ABD-R1, R1-8, R8-15, R15-16, R16-23, R16-23 del41, R23, Hinge 2-R24) were incubated with GST-Larp4 immobilized on glutathione beads in the same fashion and bound protein was detected by western blotting against His-tag. To mimic a mechanically active (open) FLNA, we used a del41 mutant whose 41 amino acid residues, which contain strand A of R20, are deleted, thereby constitutively exposing the CD face (Figure 2B). We found that full length Larp4 specifically and directly interacts with FLNA R16-23 del41, suggesting that opening R21 by mechanical force facilitates the interaction of FLNA with Larp4 (Figure 2C).

To investigate if full-length FLNA interacts with Larp4/LARP4, we expressed streptavidin-binding protein (SBP)-tagged FLNA in HEK293A cells and expressed protein was pulled down with streptavidin-beads. The beads decorated with SBP-FLNA was incubated with cell lysate of hsSKM and MEF cells. Bound Larp4/LARP4 was detected by western blotting using anti-LARP4 antibody. As expected, WT FLNA did not pulldown Larp4/LARP4, whereas del41-FLNA pulled down endogenous Larp4/LARP4 in MEF and hsSKM cell respectively (Figure 2D), suggesting that mechanically open FLNA interacts with full-length Larp4/LARP4.

### FLNA interacts with LARP4 RNA recognition motif

**FIGURE 3:**
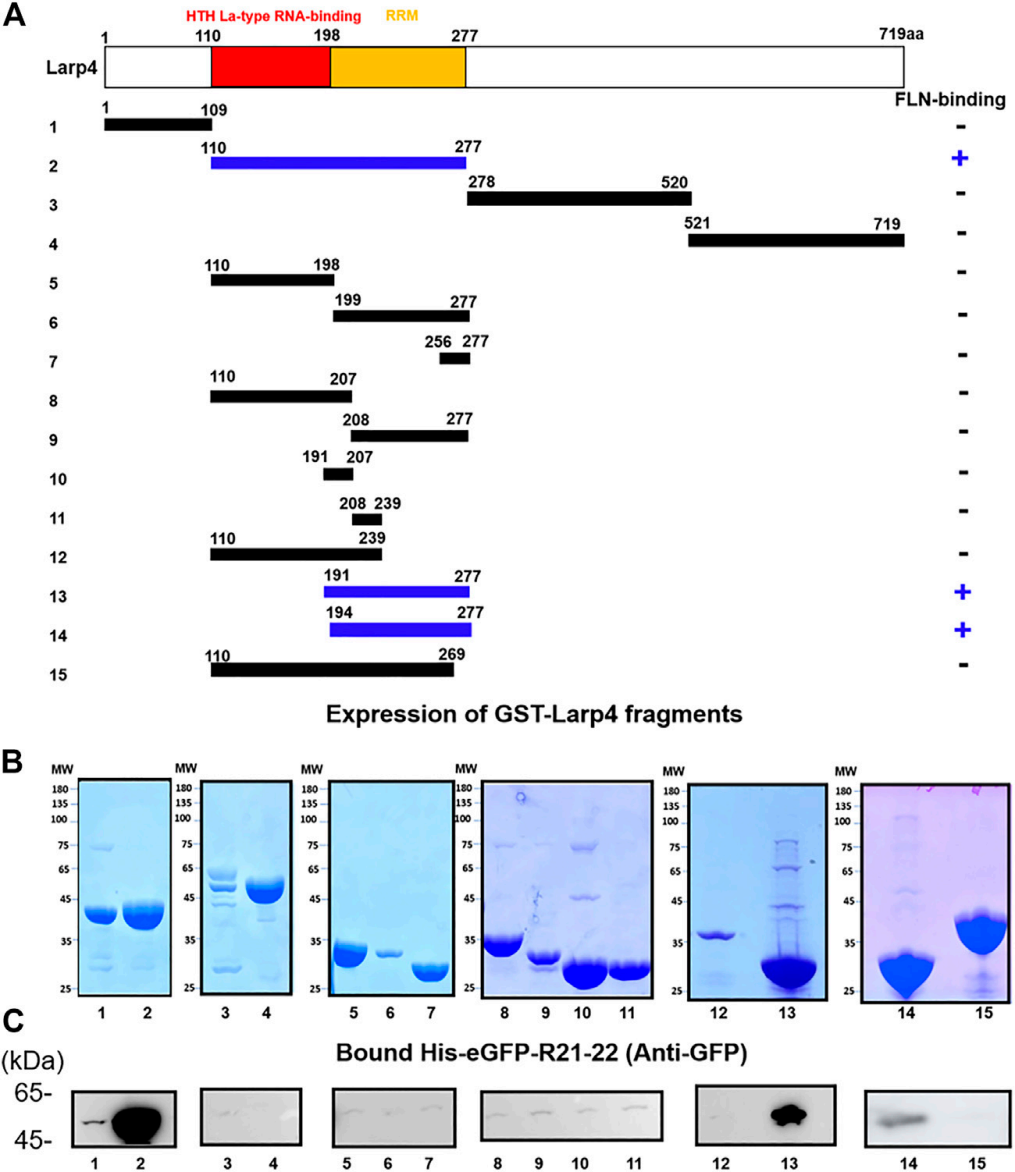
Schematic structure of Larp4 and identification of FLNA-binding domains on Larp4. **(A)** Structure of Larp4. RRM: RNA Recognition Motif. Blue indicates fragments of Larp4 that bind to FLNA R21. **(B)** CBB stained gel of 5 μg of the purified GST fusion protein. **(C)** GST-Larp4 fragments incubated with 2 μM purified His-eGFP-FLNA R21-22. Bound protein was detected by western blotting using rabbit anti-GFP antibodies.

To further narrow down the FLNA-binding site of Larp4, 15 fragments of mouse Larp4 were constructed based on its domain structure (Figure 3A). These fragment proteins were expressed as GST fusion protein, coated onto glutathione beads, and then incubated with purified His-eGFP-R21-22 (Figure 3B). Bound protein was detected by western blotting using anti-GFP antibody. We found that 110-277aa bind to FLNA R21-22 but not 110-269aa, suggesting that the FLNA binding site is located in 270-277aa (Figure 3C). However, this region is necessary but not sufficient because deletion of 191-198aa abolished the interaction. These results identified FLNA-binding site in 191-277aa that contains Larp4 RNA recognition motif.

**FIGURE 4:**
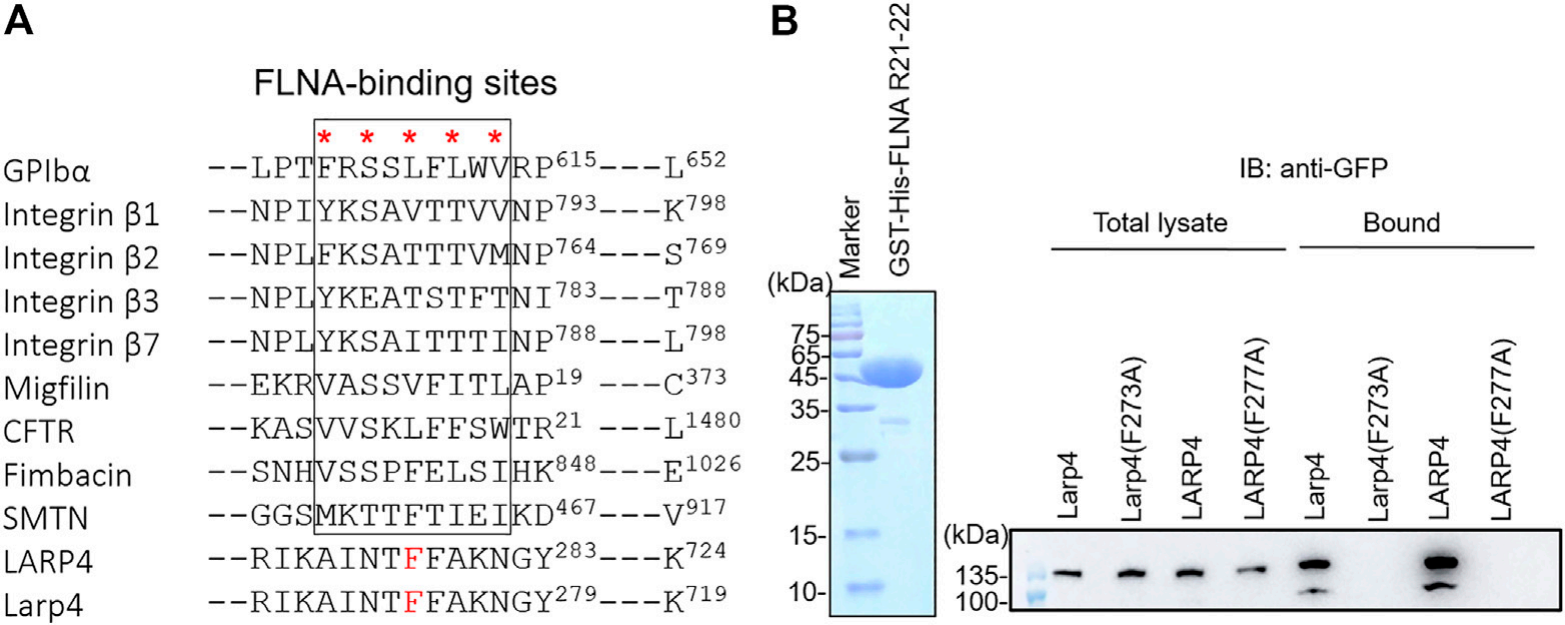
Identification of critical amino acids of LARP4 for FLNA interaction. **(A)** Alignment of binding interfaces of FLNA-binding partners. Point mutations of the underlined amino acids of mouse Larp4 (F273) and human LARP4 (F277) to Ala were predicted to disrupt the interaction with FLNA R21. **(B)** GFP-LARP4 (Larp4) (WT, F277A in LARP4 and F273A in Larp4) were expressed in HEK293A cells. Cell lysate were mixed with GST-His-FLNA R21-22 immobilized on glutathione beads. CBB staining showed 2 μg of the purified Gst-His-FLNA R21-22 protein loaded to the 15% SDS-PAGE gel. Bound GFP-LARP4(Larp4) was detected by western blotting using anti-GFP antibodies.

Earlier studies have shown that FLNA binding partners form a *β*-strand to fit into the CD face of FLNA R21. Interestingly, all structurally characterized binding partners use similar hydrophobic amino acids to interact with the CD face (FLNA-binding motif) (Figure 4A). Consistent with this alignment and the previous structural analysis of other binding partners, point mutation of F273 of mouse Larp4 and F277 of human LARP4 to Ala disrupted the interaction, demonstrating that phenylalanine is the critical amino acid for the interaction (Figure 4B). Furthermore, amino acid residues of the FLNA-binding site are highly conserved in different species of LARP4 (Supplementary Figure S2).

### FLNA specifically interacts with LARP4 but not with other LARP family proteins

**FIGURE 5:**
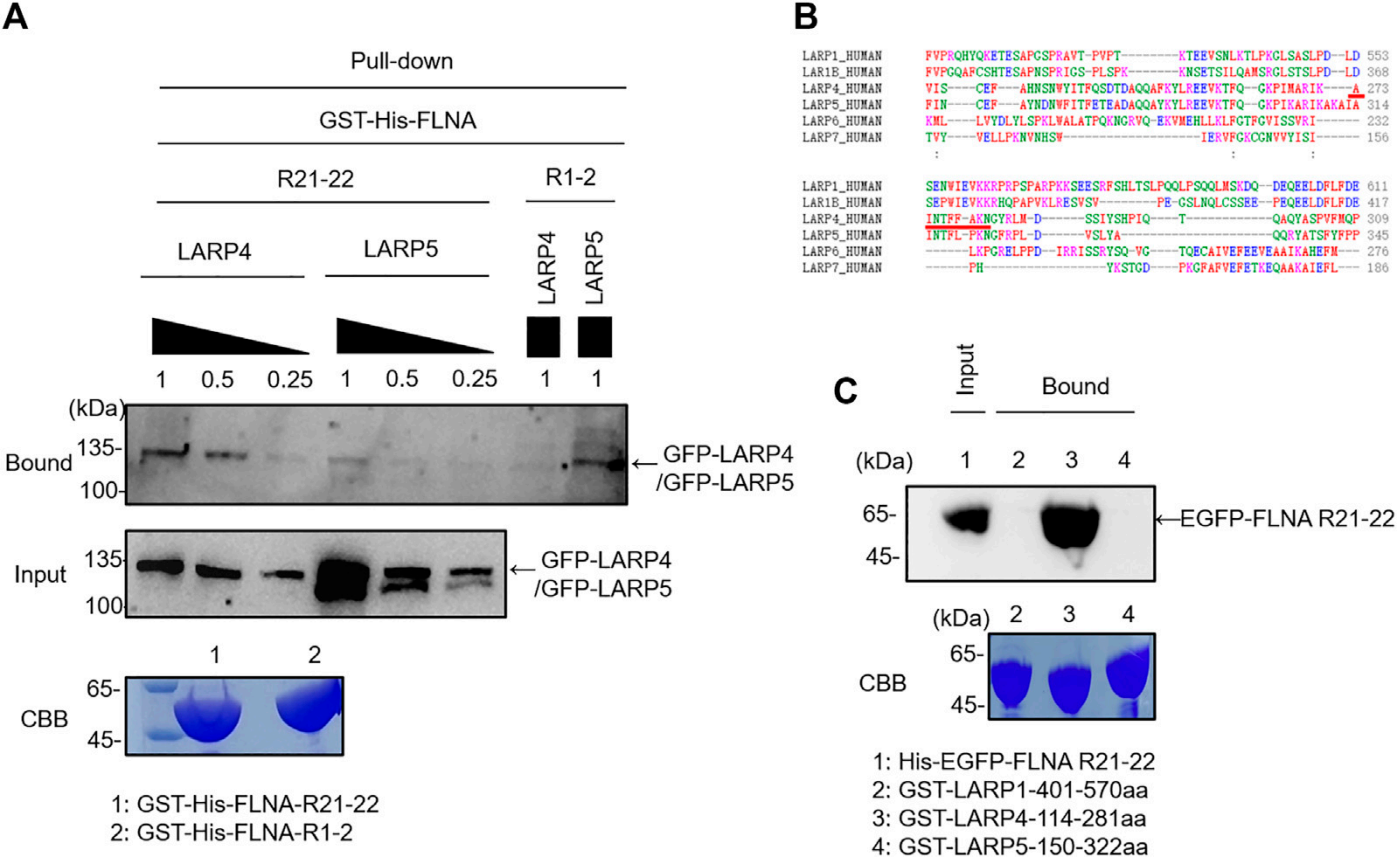
FLNA specifically interacts with LARP4 but not with other LARPs protein. **(A)** GFP-LARP5 and GFP-LARP4 were expressed in HEK293A cells and comparable expressed protein was pulled down with GST-His-FLNA R21-22 or R1-2. Bound protein was detected by western blotting using rabbit anti-GFP antibodies. **(B)** Sequence alignment of the potential FLNA-binding motif in different LARP family proteins. Red underline indicates LARP4 FLNA-binding motif. **(C)** Corresponding La modules of LARP1, LARP4 and LARP5 were expressed as GST fusion protein and incubated with 2 μM purified His-eGFP-FLNA R21-22. Bound protein was detected by western blotting using rabbit anti-GFP antibodies. 5 μg of the purified GST fusion protein was loaded to the 9% SDS-PAGE gel for CBB staining.

La-related proteins (LARPs) share a common RNA recognition unit called ‘La module’, and this module was originally found in the La protein which composed of a La motif (LaM) and an RNA recognition motif (RRM) (Alfano et al., 2004; Maraia et al., 2017). The LARPs family consists of LARP1, LARP1B, LARP3 (SSB/genuine La protein), LARP4 (LARP4A), LARP5 (LARP4B), LARP6, and LARP7. Among them, LARPs 1, 4 and 6 are highly divergent and express in the cytoplasm while LARP3 and LARP7 are nuclear protein. Given the evidence that 1) LARPs 1 and LARP1B, LARP4 and LARP5 share high homology of the amino acid sequence in the La module, 2) LARPs 1 and 4 both exhibit 3ʹ poly(A) tail length protection-mRNA stabilization (Yang et al., 2011; Aoki et al., 2013), we wondered if LARP1 and LARP5 interact with FLNA. GFP-LARP5 and GFP-LARP4 were expressed in HEK293A cells, but the expression level of LARP5 was much higher than that of LARP4 in HEK293 cells presumably because the LARP4 mRNA contains a translation-dependent, coding region determinant (CRD) of instability that limits its expression (Mattijssen et al., 2017). Thus, we used different concentration of the cell lysate to obtain a comparable level of expressed proteins and the lysates were incubated with the purified GST-His-FLNA R21-22 immobilized on glutathione beads. We found that LARP5 is not capable to interact with FLNA R21, presumably because there is a proline residue in the potential FLNA-binding site of the LARP5, which provides a proline kink that disfavoring forming a *β*-strand (Figures 5A, B). Since we found that FLNA interacts with LARP4 RNA recognition motif, the La module fragments of LARP1, LARP4 and LARP5 were expressed as GST fusion protein and immobilized on glutathione beads. The beads were incubated with purified His-eGFP-R21-22 and bound proteins were detected by western blotting against GFP. As shown in Figure 5C, LARP4 exclusively interacted with FLNA R21, demonstrating that interaction of FLNA with members of the LARPs family is specific for LARP4.

### Interaction of LARP4 with FLNA in living cells

**FIGURE 6:**
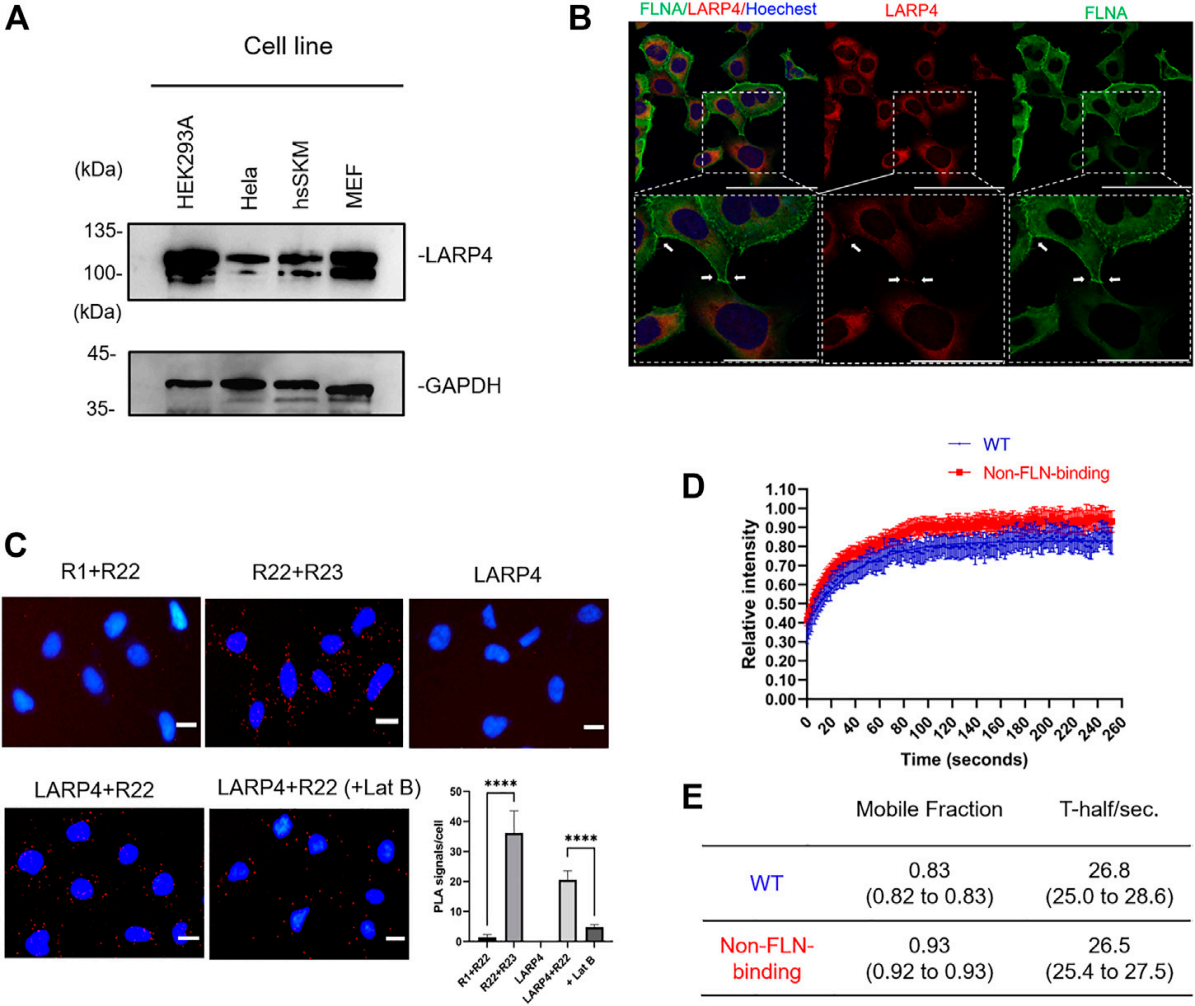
Interaction of LARP4 with FLNA in living cells. **(A)** Approximately 1.2 × 10^6^ HEK293A, Hela, hsSKM, and MEF cells cells were analyzed by WB using anti-LARP4 antibodies. The lower band is the indicative of the degradation of LARP4 protein. GAPDH is used as a loading control. **(B)** Co-localization of LARP4 and FLNA in HEK293A (Bar: 100 μm). Enlarged images are shown in the dotted box in white. Bar: 50 μm. White arrows indicate the cell-cell junction. **(C)** Interactions of LARP4 with FLNA R22 in HEK293A cells visualized by proximity ligation assay (PLA). Representative PLA images where the PLA signal (red) represents close proximity (<40 nm) between two proteins. PLA signal is significantly decreased when cells are treated with 5 μM Latrunculin B for 1 h. Graph shows the quantification of PLA between FLNA R1 and R22, R22 and R23, LARP4, LARP4 and R22 and under Lat B treatment respectively (*n* = 5). The nucleus was stained by Hoechst (blue). Scale bars are 20 μm ****p < 0.0001 was determined by the two-tailed unpaired Student’s test. **(D)** FRAP analysis was performed in HEK293A cells transfected GFP-LARP4 WT or GFP-LARP4 F277A. Quantitative analyses of FRAP assay results. Curves depict mean values (±SD) from measurements of at least 6 representative cells (n ≥ 10). WT (blue), non-FLN-binding F277A (red). **(E)** F_max_, which represents the mobile fraction of the molecule in the bleached region, and τ_½_, which is the time to recover half of the maximum fluorescence and is inversely correlated to the diffusion coefficient were analyzed by one-phase association model plugged into the Prism software. (95% confidence intervals).

The expression of Larp4/LARP4 in HEK293A, Hela, hsSKM, and MEF cells were determined by western blotting using anti-LARP4 antibody. Larp4/LARP4 is ubiquitously expressed in these cell lines (Figure 6A). Note that the lower band is likely the degradation of Larp4/LARP4 protein. Presumably different cells have different turnover cycles of Larp4/LARP4 protein. To determine whether LARP4 colocalizes with endogenous FLNA in HEK293A and hsSKM cells, we performed double staining in HEK293A and hsSKM cells with anti-FLNA and anti-LARP4 antibodies. The co-localization analysis demonstrated that these two proteins colocalize predominantly in the cytosol in HEK293A and hsSKM cell (Figure 6B; Supplementary Figure S3), suggesting that FLNA and LARP4 interact in cells. Furthermore, given the evidence that FLNA is enriched at the leading edge of the cell and the cell-cell contact (Klaile et al., 2005; Feng et al., 2006), immunofluorescence confocal microscopy of HEK293A cells demonstrated that both LARP4 and FLNA are present at the cell-cell junction (Figure 6B).

Next, *in situ* proximity ligation assays (PLA) were carried out to visualize the association of endogenous LARP4 and FLNA in cells. We raised FLNA R1, R22, and R23 specific rabbit polyclonal antibodies, affinity purified, confirmed the specificity of these antibodies (Supplementary Figure S4) and directly labeled PLA probes. Since FLNA rod-1 is longer than 50 nm (Nakamura et al., 2007) and PLA signal is usually detected when the two probes are in close proximity (<40 nm), combination of anti-R1 and anti-R22 antibodies was used as a negative control and combination of anti-R22 and anti-R23 antibodies was used as a positive control. Notably, we detected distinct PLA signals in HEK293A cells with combination of anti-LAPR4 and anti-R22 antibodies, whereas these signals have significantly reduced in LARP4-KD cells (Figure 6C; Supplementary Figure S5). Treatment of cells with latrunculin B (Lat B) to depolymerize actin filaments resulted in a significant reduction in PLA signals.

We also performed FRAP assay to determine dynamics of LARP4 in living cells (Supplementary Figure S6). FRAP assay demonstrated that the extent of recovery of fluorescence intensity of the WT GFP-LARP4 after photobleaching was significantly lesser than that of mutant LARP4 (F277A) (Figures 6D, E). However, both the WT and non-FLNA-binding LARP4 showed the similar half-life, presumably due to overexpression of GFP-LARP4. These data also demonstrated that the interaction of FLNA-LARP4 occurs in living cells.

### RNA-seq analysis of LARP4 KD cells predicts involvement of LARP4 in cell migration and morphogenesis

**FIGURE 7:**
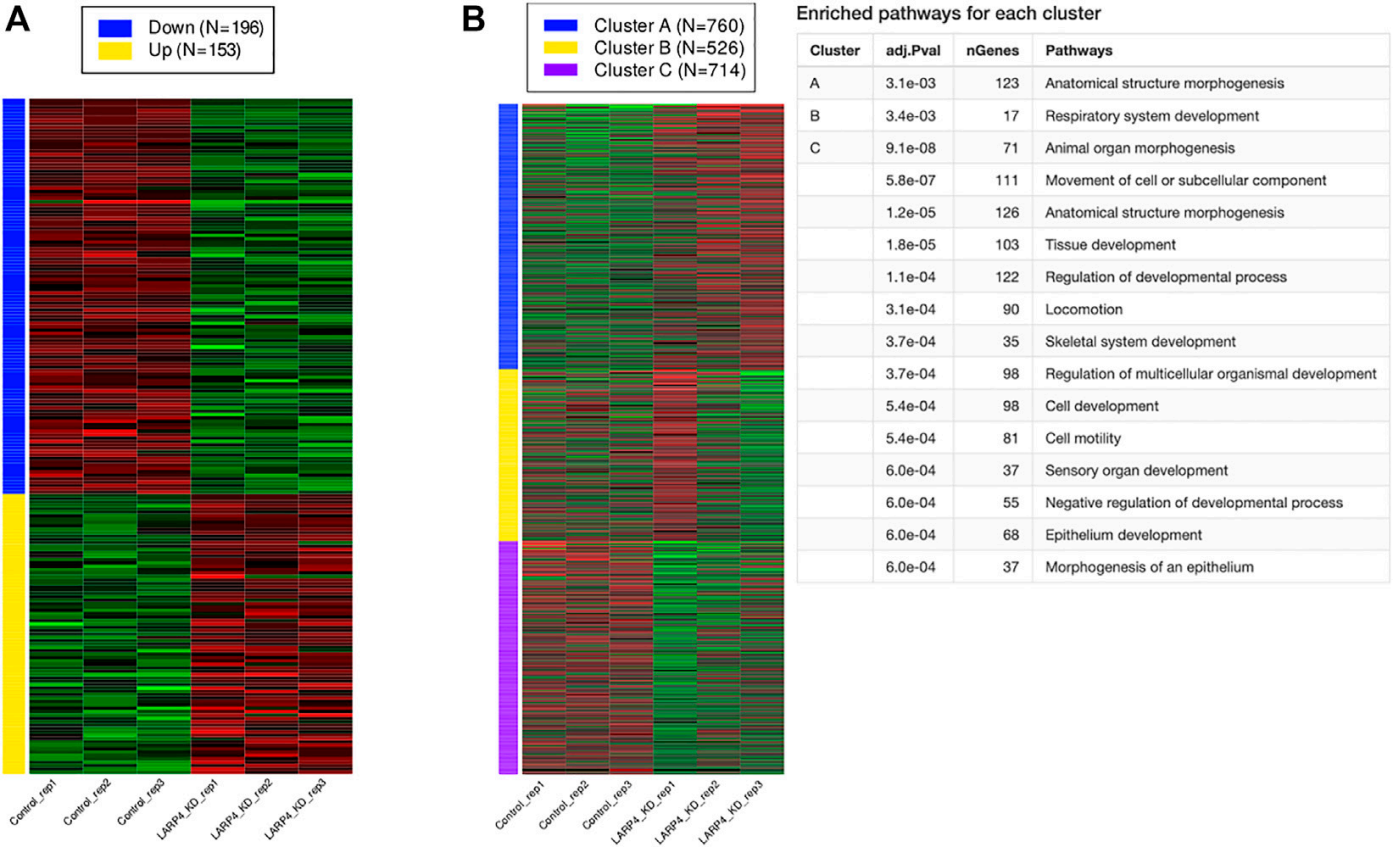
KD of LARP4 related to cell migration and morphogenesis. Differentially expressed genes (DEGs), K-means clustering, and enrichment analysis. **(A)** Numbers and expression patterns of DEGs in HEK293T cells transfected with control or LARP4 siRNA among 15,070 genes (GSE139548). **(B)** K-means clustering and enrichment analysis.

To predict the role of LARP4 in cells, we used publicly available RNAseq data to analyze the global gene expression profiles of LARP4-KD cells (Figure 7; Supplementary Figure S7). Total reads of all of the three data are reasonably high and the variation among the samples are small (Supplementary Figure S7). All genes were ranked by changes induced by gene silencing. The top 2,000 genes divided into 3 groups using *k*-means clustering according to the within-group sum of squares plot. Figure 7B shows three gene clusters and the enriched pathways for each cluster, indicating that KD of LARP4 uniquely downregulate genes in these clusters related to cell and tissue morphogenesis and cell motility. Specifically, enrichment analysis revealed that 56 genes are involved in the regulation of cell migration and locomotion upon LARP4 KD (Supplementary Data Sheet S1 **)**. Among these genes, such as *SPEF1*, *KANK1*, *CORO1B* and *EVL,* are known to positively or negatively regulate the cell migration (Cai et al., 2005; Mouneimne et al., 2012; Gee et al., 2015; Tapia et al., 2019).

### Interaction of FLNA and LARP4 is responsible for cell migration

**FIGURE 8:**
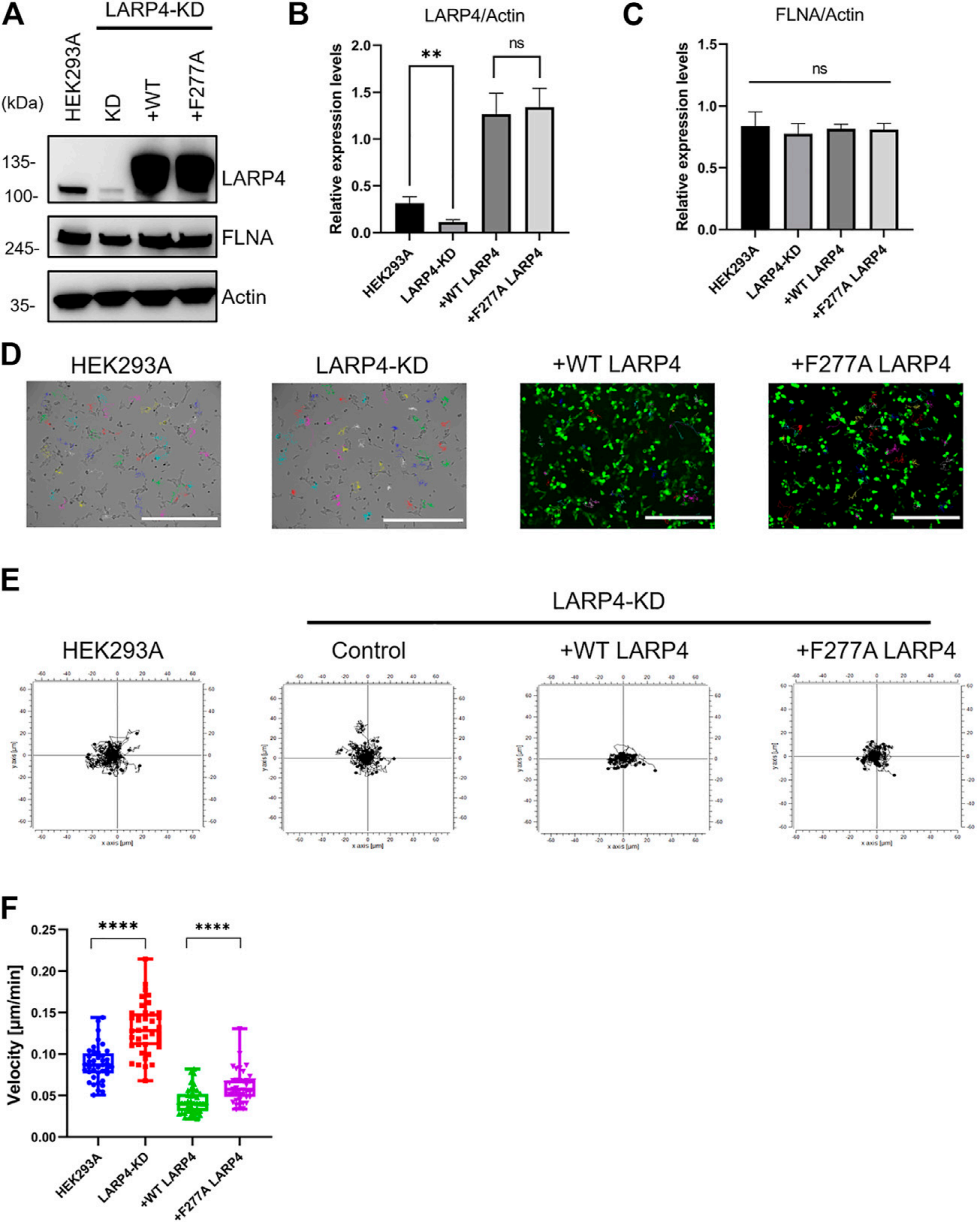
Interaction of FLNA and LARP4 inhibits cell migration. LARP4-KD HEK293A cells were transfected with GFP WT LARP4 and F277A LARP4. **(A)**. Western blot analysis for LARP4 and FLNA in LARP4-KD cells 24 h after transfection of WT and mutant GFP-LARP4. *β*-Actin was used as a loading control. Relative expression level of LARP4 **(B)** and FLNA **(C)** to actin were quantified and are shown in the diagrams with their standard deviations (*n* = 3). *p* > 0.05 was considered as not significant (ns) and **p ≤ 0.01 were determined by two-tailed unpaired Student’s *t*-test or one-way ANOVA analysis. **(D)**. Images of cells were captured at 1 frame/10 min for 16 h by time-lapse microscopy and tracked the migratory paths of cells (*n* = 40). Bar: 400 μm. **(E)** Plots show an example tracking 40 cells from one of three independent experiments. **(F)** Mean migration speed (μm/min) of cells. Values represent the migration speed of each cell as a dot. ****p < 0.0001 was determined by the two-tailed unpaired Student’s test.

To investigate the biological function of LARP4-FLNA interaction in cells, *LARP4* gene was edited in HEK293A cells by the CRISPR-Cas9 system. A marked decrease in the level of LARP4 (70.0% ± 6.3%) was observed upon gene editing of *LARP4* compared with WT HEK293A cells (Figures 8A, B). WT and mutant GFP-LARP4 were added back to LARP4-KD HEK293A cells at a comparable level, while no significant different of the FLNA expression level was observed (Figures 8B, C). Because FLNA is responsible for cross-linking actin filaments into orthogonal networks (Nakamura et al., 2007), we next examined the role of LARP4-FLNA interaction on the actin cytoskeleton structure using HEK293A WT and LARP4-KD cells by confocal microscopy. The phalloidin staining images and 3D structure showed no apparent difference between WT and KD cells (Supplementary Figure S8; Supplementary Movie S1, S2), suggesting that interaction of LARP4-FLNA does not affect the structure of the actin filaments networks.

Since both FLNA and LARP4 play an important role in cell migration (Klaile et al., 2005; Zhong et al., 2010; Kircher et al., 2015; Seetharaman et al., 2016; Chiang et al., 2017), we compared the migration of cells expressing WT and mutant LARP4 using the LARP4-KD cells (Figure 8D and Supplementary Movie S3, S4). Consistent with the previous report (Seetharaman et al., 2016; Egiz et al., 2019), we confirmed that LARP4-KD HEK293A cells migrated faster than WT HEK293A with a significant 2-fold increase in cell migration speed in random orientation (Figures 8E, F). Importantly, LARP4-KD cells expressing mutant LARP4 also resulted in a significant increase in cell migration speed compared to the LARP4-KD cells expressing WT LARP4 (Figures 8E, F). Collectively, we provide evidence that the interaction between FLNA and LARP4 inhibits cell migration.

## Discussion

Difficulty to maintain mechanical force in lysed cells and reconstituted systems is the major obstacle in mechanotransduction research. After lysing cells, force-dependent FLNA-partner interaction will be lost, making it difficult to carry out conventional binding assays. Therefore, we rationally designed a probe that mimics mechanically activated FLNA molecule and identified LARP4 that binds to FLNA mechanosensing domain R21. Previous studies show that cleavage of FLNA by the calcium-dependent protease calpain in Hinge 1 produces a 110 kDa C-terminal fragment (FLNA^CT^, R16-24) and this fragment is further cleaved to a 90 kDa fragment (R16–23), which can translocate to the cell nucleus (Fox et al., 1985; van der Flier and Sonnenberg, 2001; Mooso et al., 2012). However, our results indicated that there was no cleavage of the FLNA when interacts with LARP4 because binding of LARP4 did not reduce the amount of the full-length FLNA del41 compared to the WT FLNA (Figure 2D). Double staining of LARP4 and FLNA confirmed their colocalization in the cytosol in HEK293A cells and cell-cell contact site, suggesting that the FLNA-LARP4 interaction occurs in the cytoplasmic region. PLA and FRAP assays also confirmed their interaction in cells.

The FLNA binding site of LAPR4 contains a typical FLNA-binding motif that was predicted by the alignment of the binding sites of known FLNA-binding proteins. Mutagenesis of the amino acids on the binding sites disrupted the interaction, indicating that LARP4 interacts with FLNA R21 in the same fashion as other FLNA R21 binding partners. These findings suggested that the FLNA-LARP4 interaction occurs in living cells under mechanical stress. However, how R21 interact with LARP4 at atomic level is not known although the structure of LARP4 is already reported (Cruz-Gallardo et al., 2019). Thus, further study is necessary to reveal the molecular structure of the LARP4-R21 complex.

Consistent with previous finding of LARP4 function (Seetharaman et al., 2016), RNA-seq analysis of LARP4-KD cells indicated the involvement of LARP4 in cell migration. RNA-seq analysis also suggested LAPR4 regulates cell morphogenesis and development in various tissues, presumably because cell migration controls these biological processes. Moreover, RNA-seq analysis suggested how LARP4 regulates cell migration. For example, among these downregulated genes related to cell migration by LARP4 KD, SPEF1 (Sperm flagellar 1) has been reported as an actin-binding protein to organize actin cytoskeletons in lamellipodia and filopodia at the leading edge of migrating cells (Tapia et al., 2019). Further experiments need to be performed to investigate the genes regulated by LARP4 on cell migration and other functions as suggested by RNA-seq analysis.

We generated the LARP4-KD HEK293A cells with 70.0% ± 6.3% KD efficiency by CRISPR-Cas9 system, while previous studies used siRNA-mediated KD of LARP4 (Seetharaman et al., 2016). Consistent with the previous report, we also confirmed that LARP4 suppresses cell migration, as the migration speed increased upon KD of LARP4 (Seetharaman et al., 2016; Egiz et al., 2019). As expected, in our transient transfection experiments, we observed a significant decrease in cell migration speed upon expression of WT LARP4 in LARP4-KD cells. Conversely, expressing the F277A mutant LARP4 abrogates this inhibitory function, suggesting that the interaction of LARP4 with FLNA regulates cell migration. Although we cannot exclude the possibility that F277 is involved in other cellular processes than binding to FLNA, there is no another mean to specifically perturb the interaction. Since FLNA R21 binds to multiple partners, such as integrin, migfilin and G3BP1 (Lad et al., 2008; Ehrlicher et al., 2011; Feng et al., 2023), it is also possible that the disruption of the FLNA-LARP4 interaction interfere the functions of these partners and thus regulates cell migration. However, point mutation of FLNA could also influence not only LARP4 interaction but also other interactions. Therefore, we used F277A mutant LARP4 to study the function of the FLNA-LARP4 interaction in cells.

LARP4 binds to the poly(A) sequence of mRNA and is involved in multiple signaling pathways to play crucial roles in cell proliferation, migration and adhesion (Bai et al., 2011; Seetharaman et al., 2016). In addition to mRNA, the receptor for activated C kinase (RACK1) and the poly(A)-binding protein (PABPC1) have been identified as direct binding partners for LARP4 (Yang et al., 2011). These interactions could therefore promote mRNA translation and stability, 3′UTR poly(A) lengthening, post-transcriptional regulation of ribosomal process and mRNA processing. Since FLNA-binding site is located at C-terminal of the RNA recognition motif of LARP4, our results suggest an intriguing possibility that the FLNA-LARP4 interaction alters the translation of mRNAs.

The Rho GTPase family proteins including the Rho, Rac and Cdc42, are considered to play the major role in regulating cell migration and actin reorganization (Heasman and Ridley, 2008; Ridley, 2015). FLNA has been shown to bind to the Rho family GTPases and to some of their regulatory cofactors to regulate actin remodeling, formation of filopodia, and membrane ruffles (Ohta et al., 1999; Ueda et al., 2003; Nakamura, 2013). Interestingly, a recent study found that LARP4 depletion was associated with the increase in RhoA protein expression, suggesting that LARP4 may limit RhoA-dependent cell motility (Egiz et al., 2019). Thus, we could not rule out the possibility that LARP4-FLNA interaction suppresses RhoA activity while the dissociation of LARP4 from FLNA leads to increasing of the RhoA activity and consequently regulate the cell migration through the Rho signaling networks (Narumiya et al., 2009; Bai et al., 2011; Vega et al., 2011; O'Connor and Chen, 2013).

Seetharaman et al. (2016) demonstrated that LARP4 depletion increased cell motility and invasiveness in PC3 prostate cancer cells and MDA-MB-231 breast cancer cells. Furthermore, a recent study reported that the LARP4 mRNA-high expression group showed longer overall survival compared with the LARP4 mRNA-low expression group, implying a positive correlation of LARP4 mRNA levels in ovarian cancer tissues with patient prognosis (Egiz et al., 2019). Therefore, LARP4 could suppress motility and metastatic potential of ovarian cancer cells.

Taken together, our finding sheds new light on a mechanotransduction pathway mediated by the FLNA-LARP4 interaction to regulate cell migration, which might be a novel drug target to control cancer growth and metastasis.

## Data Availability

The original contributions presented in the study are included in the article/Supplementary Material, further inquiries can be directed to the corresponding author.
