# Supplementary material for: Interaction of LARP4 to filamin A mechanosensing domain regulates cell migrations
Source: Front Cell Dev Biol. 2023 Apr 24;11:1152109. doi: 10.3389/fcell.2023.1152109 (PMC10164935; doi:10.3389/fcell.2023.1152109)
Supplement: Supplementary file 6 [file Table2.DOCX]

Supplementary Material

**Interaction of LARP4 to filamin A mechanosensing domain regulates cell migrations**

**Zhenfeng Mao and Fumihiko Nakamura***

*** Correspondence:** Corresponding Author: Fumihiko Nakamura: School of Pharmaceutical Science and Technology: [fnakamura@tju.edu.cn](mailto:fnakamura@tju.edu.cn); Tel: +86- 22-87401830

List of Supplementary Material:

Supplementary Figures S1-S8

Supplementary Table S1

Supplementary Movies S1-S4

# Supplementary Figures and Tables

## Supplementary Figures


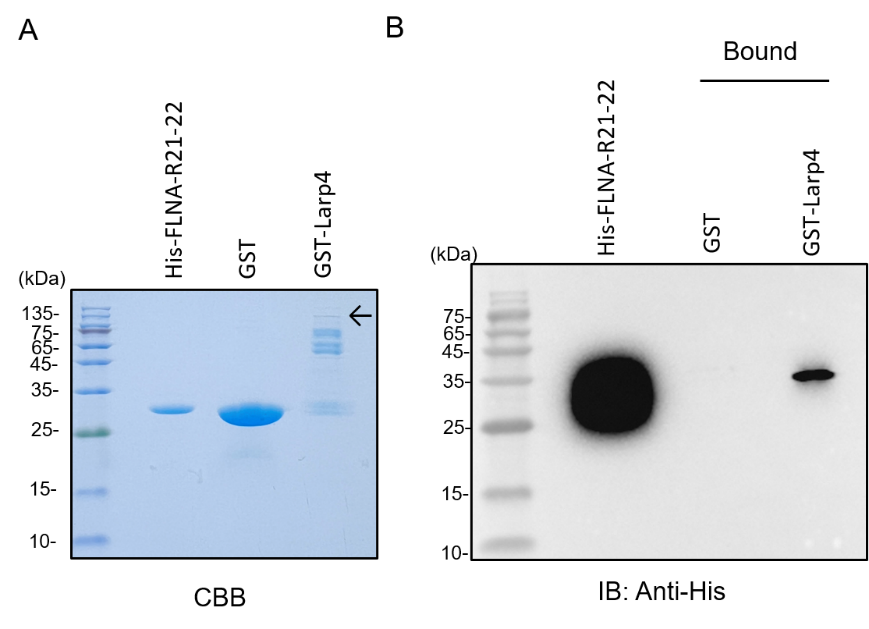


**Supplementary Figure S1**. FLNA R21-22 interacts with mouse Larp4 directly.

(**A**) CBB staining of the purified GST, GST-Larp4 and His-FLNA R21-22 protein. Black arrow indicates the GST-Larp4. (**B**) Purified His-FLNA R21-22 were pulled down with GST-Larp4 immobilized on glutathione beads. Bound His-tagged FLNA fragments were detected by western blotting using an anti-His-tag antibody.

**
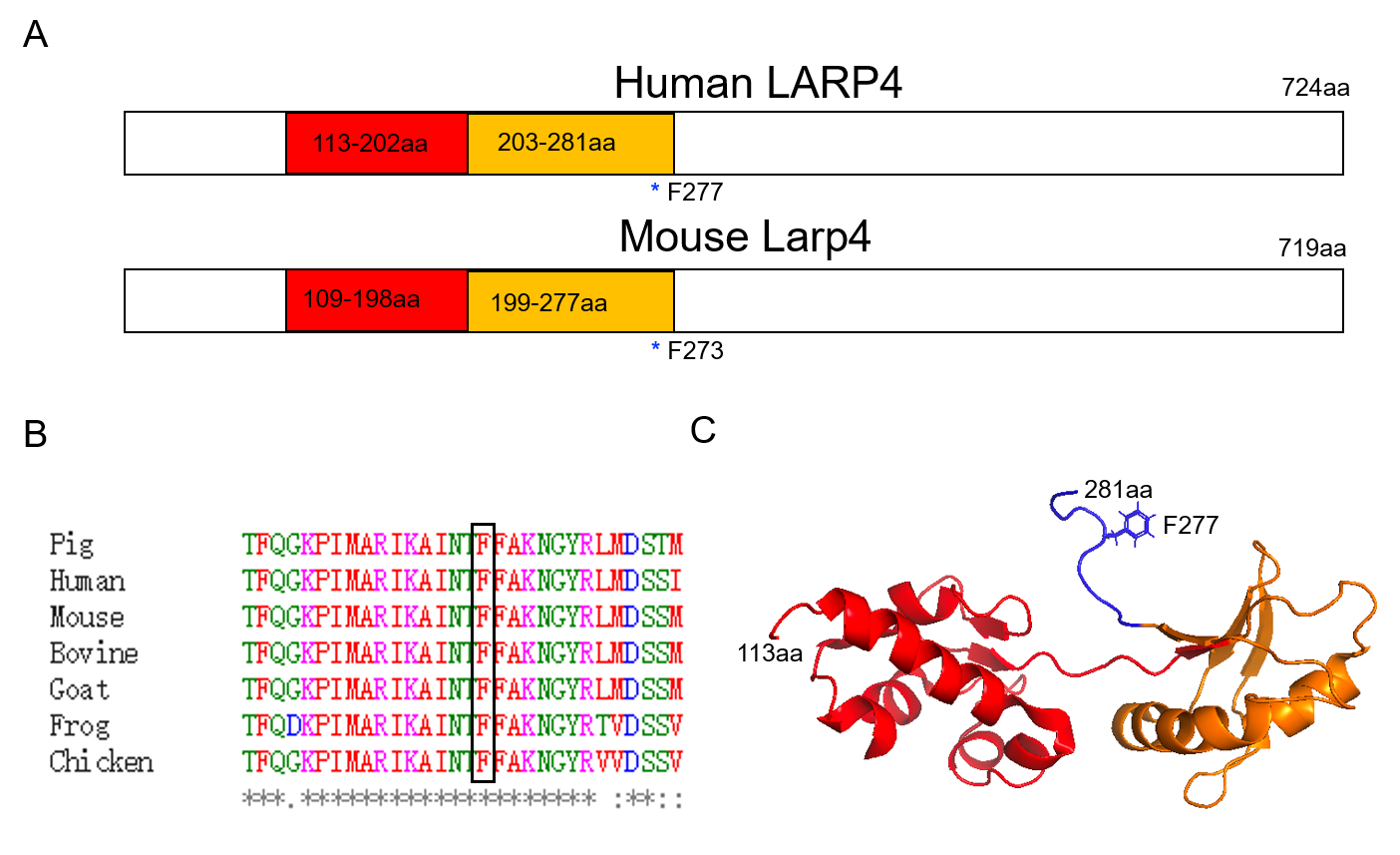
**

**Supplementary Figure S2.** Structure of human and mouse LARP4 proteins.

(**A**) Comparison of human LARP4 and mouse Larp4 proteins. HTH La-type RNA-binding (Red), RRM: RNA Recognition Motif (Orange). (**B**) Sequence alignment of the critical amino acid residue in different LARP4 species. (**C**) Structure of LARP4 domains. FLNA-binding motif is highlighted in blue showing disordered structure. F277 is critical for the FLNA interaction. Cited from PDB: 6I9B.

**
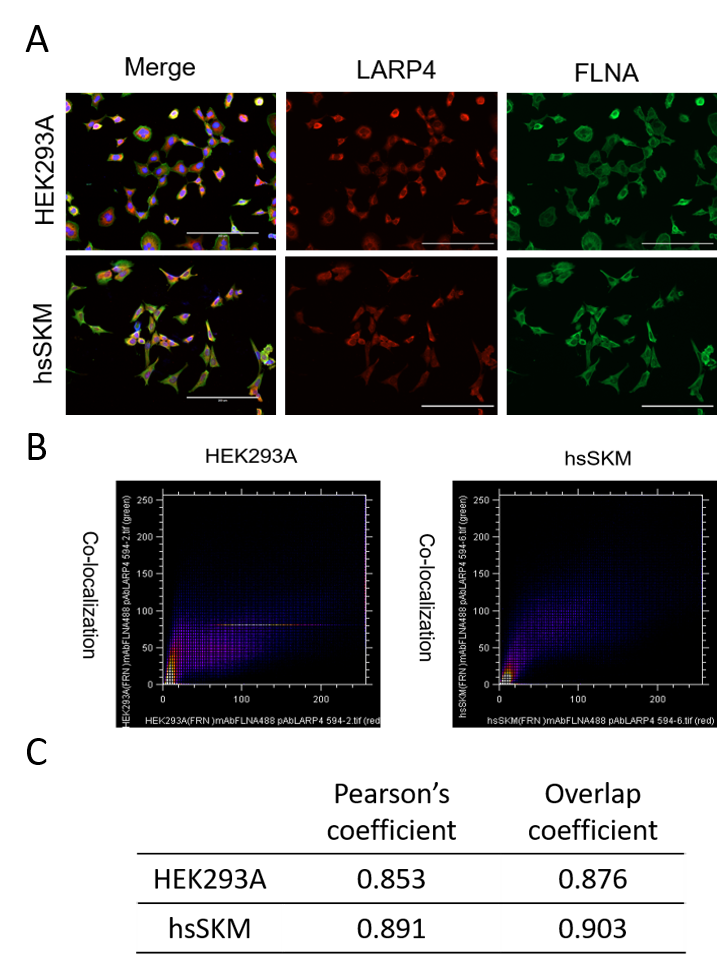
**

**Supplementary Figure S3.** FLNA and LARP4 colocalize predominantly in the cytosol in HEK293A cell and hsSKM cells. **(A)** Double staining of LARP4 and FLNA in HEK293A and hsSKM cell. Bar: 200 μm. (**B**) Colocalization was analyzed using ImageJ plug-in, Colocalization Finder. Scatterplots show the co-localization in HEK293A and hsSKM cell respectively. The intensity of the color of the scatterplot represents the frequency of its appearance. The more diagonal the scatterplot is, the higher the degree of co-localization. (**C**) Pearson’s coefficient and Manders’ overlap coefficient were calculated from the scatterplots. (n=5).


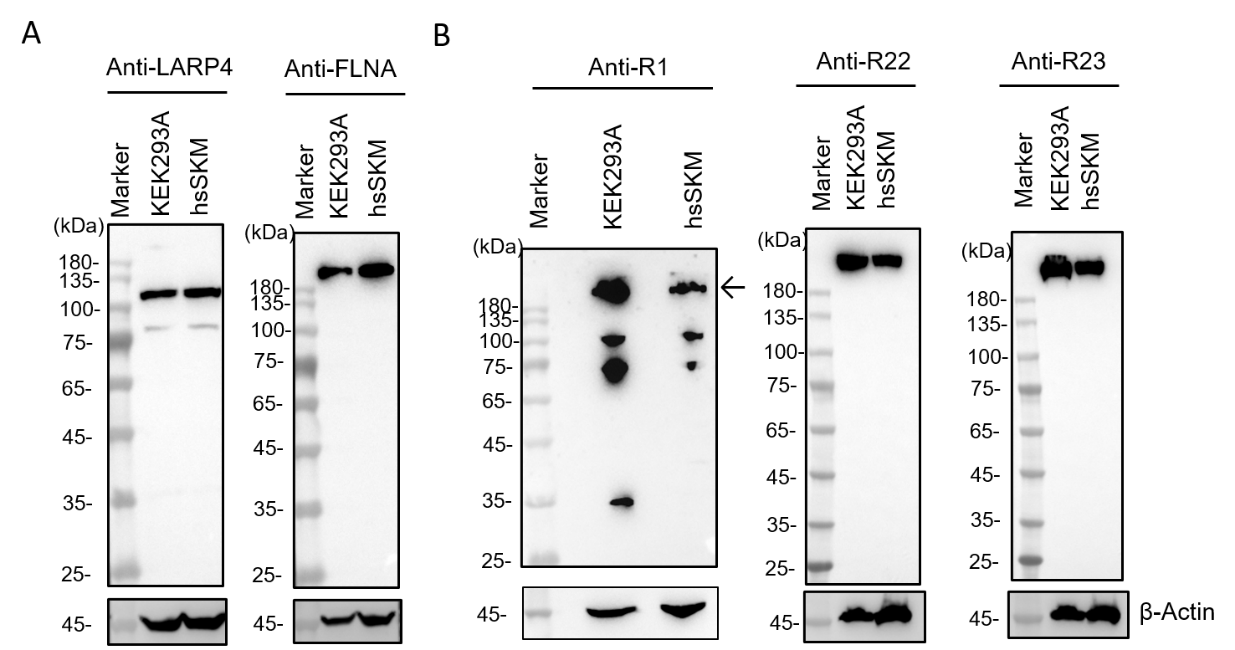


**Supplementary Figure S4.** Specificity of the antibodies used for IF and PLA. HEK293A and hsSKM cell lysate were blotted using (**A**) polyclonal anti-LARP4 and monoclonal anti-FLNA antibodies and (**B**) polyclonal anti-FLNA R1, R22 and R23 antibodies. β-Actin was used as a loading control.


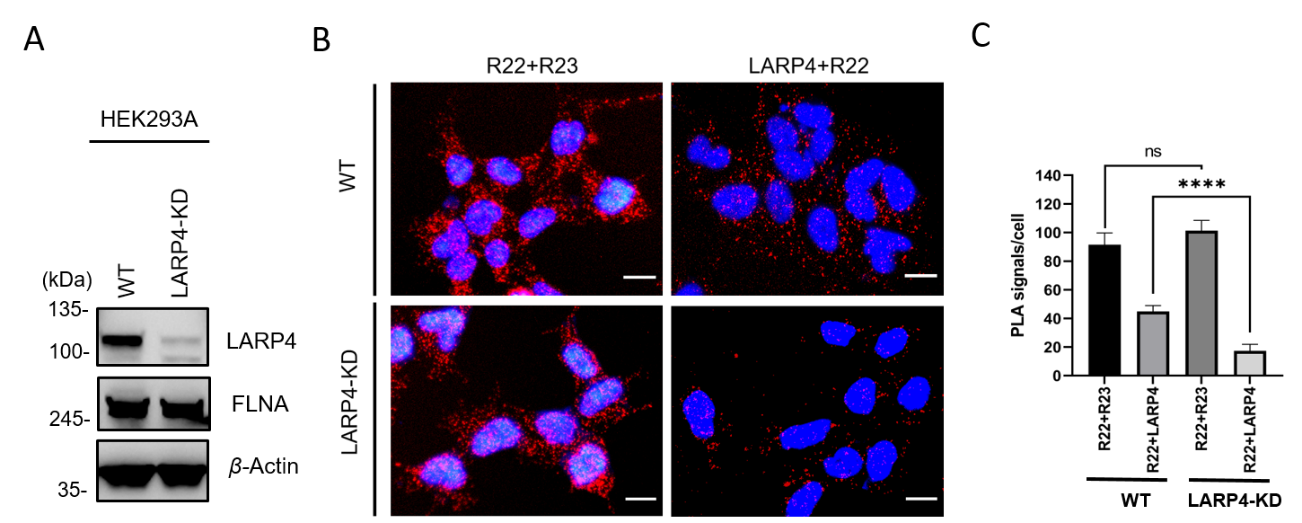


**Supplementary Figure S5.** PLA signal of LARP4- FLNA interaction is significantly reduced in LARP4-KD HEK293A cells. (**A**) Western blot analysis for LARP4 and FLNA in LARP4-KD cells. *β*-Actin was used as a loading control. (**B**) FLNA R22 interaction with R23 serving as a positive control. Images show a decreased PLA signal between LARP4 and FLNA R22 in LARP4-KD cells. (**C**) Quantification of PLA between FLNA R22 and R23, LARP4 and FLNA R22 in WT HEK293A cells and LARP4-KD cells respectively (n=6). The nucleus was stained by Hoechst(blue). Scale bars are 20 μm. ns (no significance) and ****P<0.0001 were determined by the two-tailed unpaired Student’s test.


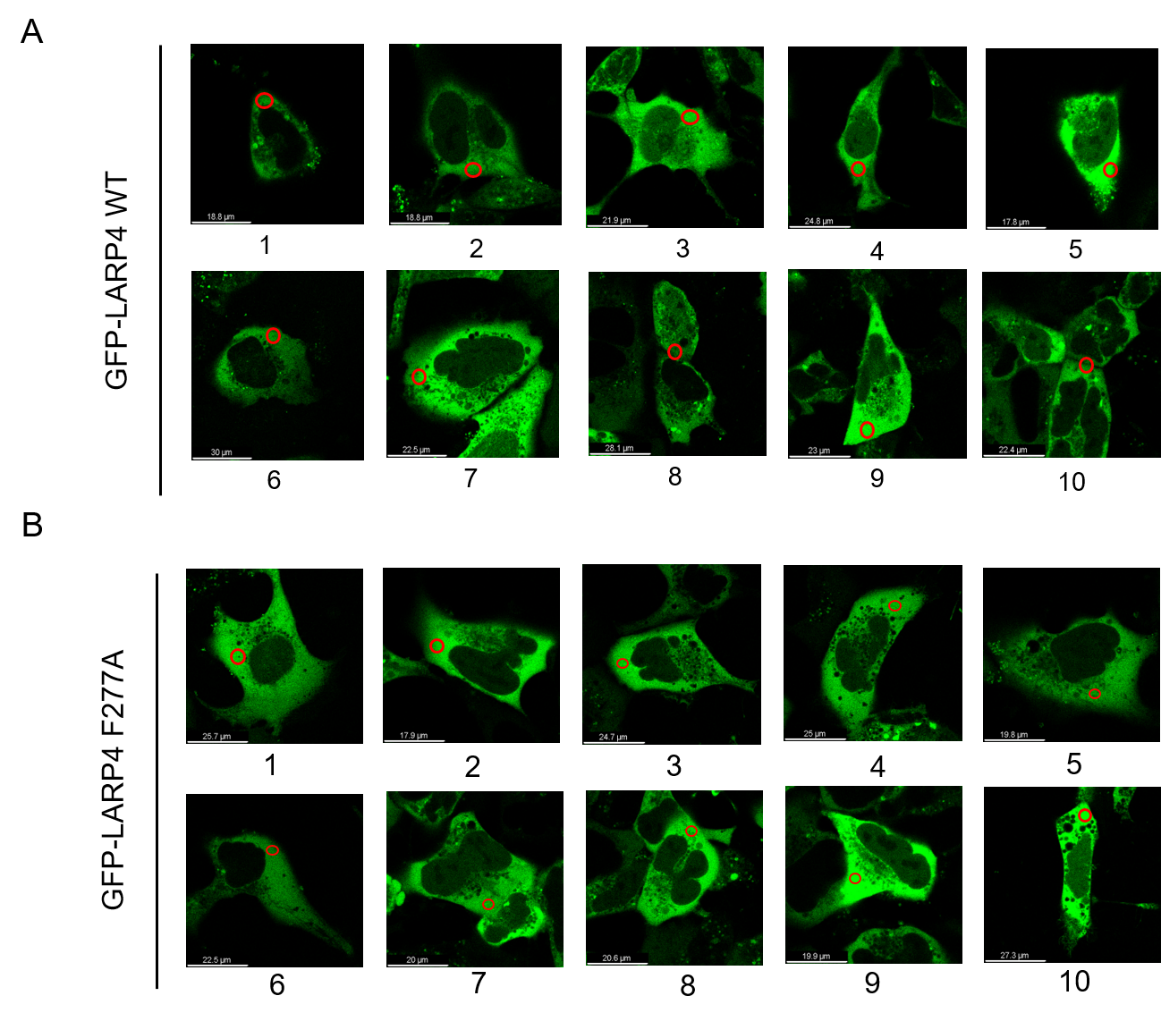


**Supplementary Figure S6.** HEK293A cells transfected with (**A**) GFP-LARP4 WT and (**B**) GFP-LARP4 F277A for FRAP analysis (n=10), red circle indicates the region for bleach, area: 3 μm×3 μm.


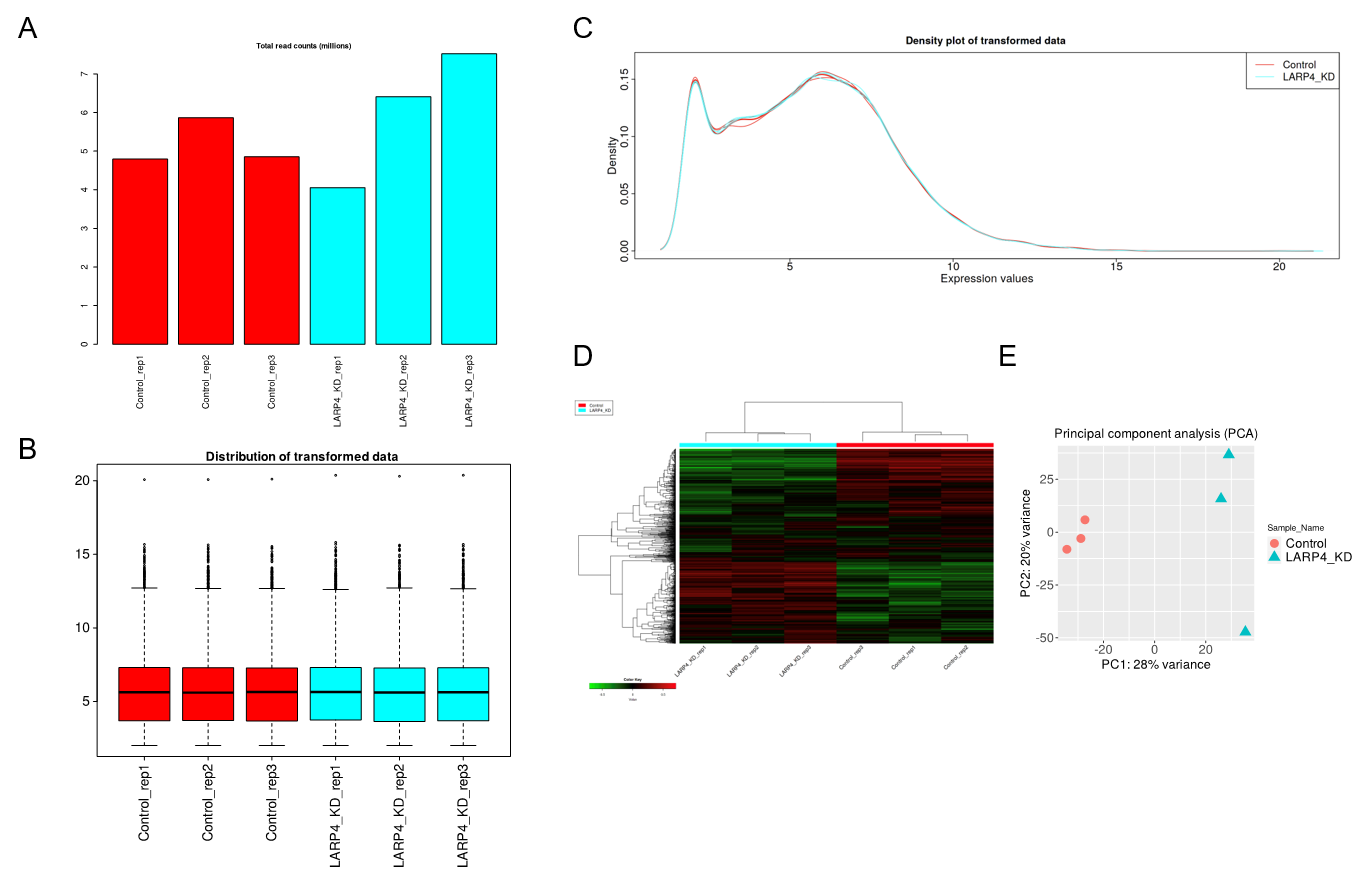


**Supplementary Figure S7.** Diagnostic plots for read-counts data. (**A**) A bar plot of total read counts per library, showing some variation in library sizes. (**B**) Distribution of transformed data using a density plot. (**C**) Boxplot of transformed data. Variation among replicates is small (**B** and **C**).  (**D**) Hierarchical clustering. (**E**)  Principal component analysis (PCA) analyses indicate the substantial difference in thousands of genes induced by LARP4 knockdown. There is some variation among replicates.


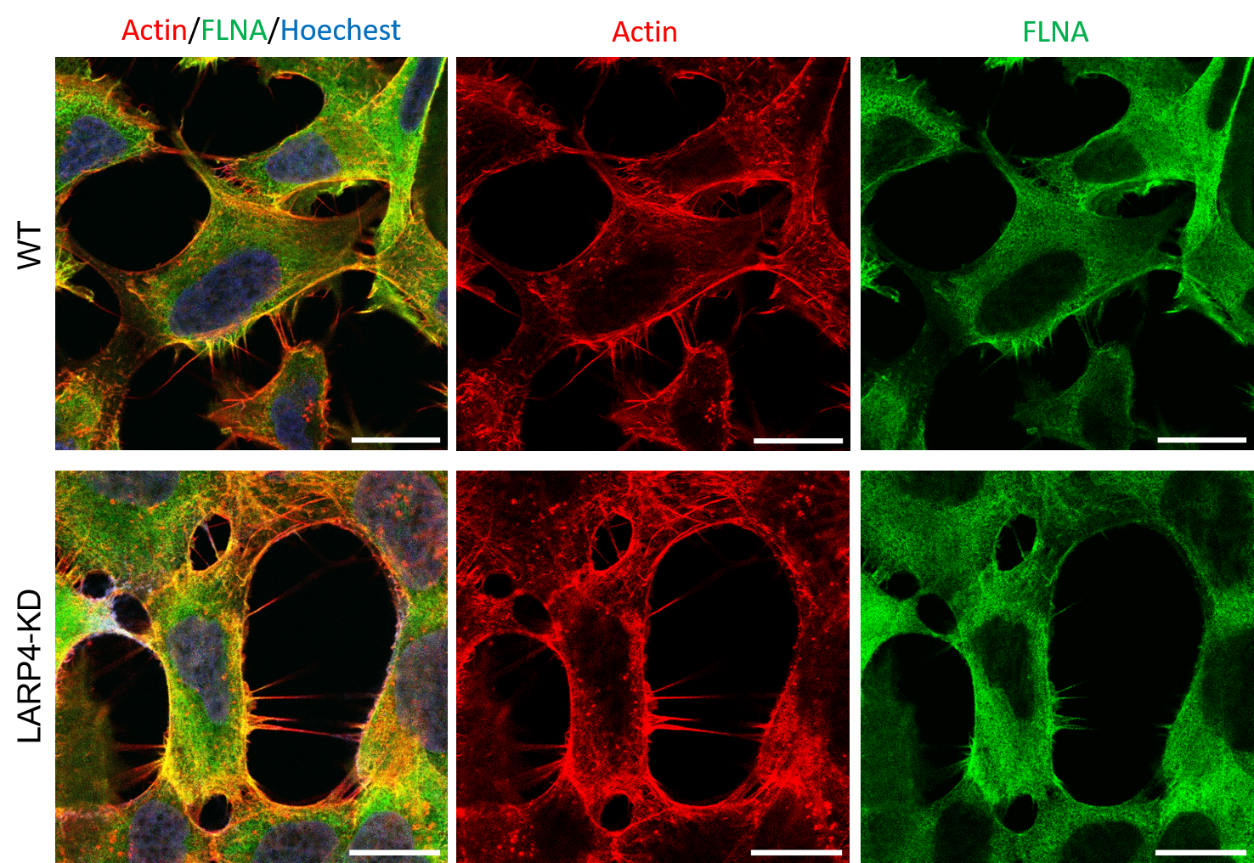


**Supplementary Figure S8.** LARP4-FLNA interaction does not affect the structure of the actin filaments networks. HEK293A cells and LARP4-KD cells were fixed and stained with Alexa-568 phalloidin, FLNA (Alexa-488) and Hoechst 33342. Images were taken by confocal microscopy using 100× objective. Bar: 25 μm.

## Supplementary Table

| **Name** | **Sequence** |
| --- | --- |
| Probe-1 | 5′ azide – AAAAAAAAAATATGACAGAACTAGACACTCTT |
| Probe-2 | 5′ azide – AAAAAAAAAAGACGCTAATAGTTAAGACGCTT - 3 × 2′ O-methyl RNA uracil (UUU) |
| Circularization oligonucleotide 1 | 5′ phosphate – GTTCTGTCATATTTAAGCGTCTTAA |
| Circularization oligonucleotide 2 | 5′ phosphate – CTATTAGCGTCCAGTGAATGCGAGTCCGTCTAAGAGA GTAGTACAGCAGCCGTCAAGAGTGTCTA |
| Detection oligonucleotide | Cy3-CAGTGAATGCGAGTCCGTCT – 3 × 2′ O-methyl RNA uracil (UUU) |

**Supplementary Table S1.** Oligonucleotides used for PLA (Söderberg, Gullberg et al. 2006)

## Supplementary Movies (See additional uploaded videos):

Movie S1:

HEK293A FLNA-488 and phalloidin-568 3D structure

HEK293A phalloidin-568 3D structure

**Supplementary Movies S1.** HEK293A cells stained with Alexa-568 phalloidin (red), FLNA (green) and Hoechst 33342 (blue). Images were taken by Leica SP8 X confocal microscope using 100x objective. 3D structure model was generated by scanning the whole cell from top to down at 700 Hz using 2048×2048 pixels format.

Movie S2:

LARP4-KD FLNA-488 and phalloidin-568 3D structure

LARP4-KD phalloidin-568 3D structure

**Supplementary Movies S2.** LARP4-KD HEK293A cells stained with Alexa-568 phalloidin (red), FLNA (green) and Hoechst 33342 (blue). Images were taken by Leica SP8 X confocal microscope using 100x objective. 3D structure model was generated by scanning the whole cell from top to down at 700 Hz using 2048×2048 pixels format.

Movies S3:

cell migration of WT HEK293A cells-Overlay Dots & Lines

cell migration of LARP4-KD cells-Overlay Dots & Lines

**Supplementary Movies S3.** HEK293A cells and LARP4-KD cells were seeded at 10^4^ cells per well on 6-well plates. Images were acquired for 16 h at 1 frame/10 min at 37C using a 10X Plan objective on an EVOS® FL Auto time lapse microscope with a monochrome camera. Cells were tracked using ImageJ (Plugin: Manual tracking) to obtain migration speed (μm/min). Movies showed the tracking the migratory paths of 40 cells labeled in different colors.

Movies S4:

Add back WT LARP4 of LARP4-KD cells-Overlay Dots & Lines

Add back F277A LARP4 of LARP4-KD cells-Overlay Dots & Lines

**Supplementary Movies S4.** LARP4-KD HEK293A cells were transfected with GFP-WT LARP4 and F277A LARP4. After a further 24 h, images were acquired for 16 h at 1 frame/10 min at 37C using a 10X Plan FL objective on an EVOS® FL Auto time lapse microscope with a color camera. Cells were tracked using ImageJ (Plugin: Manual tracking) to obtain migration speed (μm/min). Movies showed the tracking the migratory paths of 40 cells labeled in different colors.

**Reference:**

Söderberg, O., M. Gullberg, M. Jarvius, K. Ridderstråle, K. J. Leuchowius, J. Jarvius, K. Wester, P. Hydbring, F. Bahram, L. G. Larsson and U. Landegren (2006). "Direct observation of individual endogenous protein complexes in situ by proximity ligation." Nat Methods **3**(12): 995-1000.
